# Validity and reliability of the Japanese version of the sustainability consciousness questionnaire

**DOI:** 10.3389/fpsyg.2023.1130550

**Published:** 2023-03-15

**Authors:** Hiroyoshi Ogishima, Ayahito Ito, Shogo Kajimura, Toshiyuki Himichi

**Affiliations:** ^1^Research Institute for Future Design, Kochi University of Technology, Kochi, Japan; ^2^Department of Psychology, University of Southampton, Southampton, United Kingdom; ^3^Faculty of Health Sciences, Hokkaido University, Sapporo, Japan; ^4^Faculty of Information and Human Sciences, Kyoto Institute of Technology, Kyoto, Japan; ^5^School of Economics and Management, Kochi University of Technology, Kochi, Japan

**Keywords:** sustainability consciousness questionnaire, sustainability consciousness, sustainable development, sustainable development goals, sustainability

## Abstract

The sustainable development goals (SDGs) are required to be achieved by 2030, and measurement indicators are needed to properly visualize individual efforts toward SDGs. Here, we developed a Japanese version of the Sustainability Consciousness Questionnaire (SCQ), the most well-known individual measure of SDGs, and examined its reliability and validity. Three online surveys were conducted with 1,268 Japanese adults. The results of confirmatory factor analysis showed that the Japanese version of the SCQ consists of two single-level factors: sustainability knowingness/attitude and sustainability behavior. These two factors demonstrated sufficient internal consistency by Cronbach's alpha and McDonald's omega coefficient, which ensured measurement reliability. Additionally, cocorrelations with other scales indicated that the higher the level of sustainability knowledge and attitude, the less positive attitude toward climate change and the higher the level of sustainability behavior, indicating the construct validity of these factors. These results indicate that the Japanese version of the SCQ is reliable and valid.

## Introduction

The Sustainable Development Goals (SDGs) are international goals to achieve “a development that meets the needs of the present generation without compromising the ability of future generations to meet their own needs” (Al-Athel et al., 1987). It consists of 17 goals spanning social, economic, and environmental domains, and is structurally composed of 15 subthemes^1^ (UNESCO, 2005; Buckler and Creech, 2014). Regarding the SDGs, all United Nations member states, including Japan, aim to achieve the goals by 2030 (United Nations Department of Economic Social Affairs, 2016) and measurement indicators are needed to efficiently visualize their efforts (Miola and Schiltz, 2019). However, even though various indicators have been proposed to identify national-level efforts (e.g., United Nations, 2017; Sachs et al., 2021), few indicators have been established by which to identify person-level efforts (See for a review, Boulanger, 2008). In Japan in particular, there is a large gap between national- and person-level commitment to the SDGs. The latest national survey shows that only 29.2% of Japanese people know what the SDGs are (Ministry of Education Culture, Sports, Science and Technology, 2019). To encourage future research that ascertains public awareness of the SDGs and enlightens individuals regarding their efforts, it is necessary to establish reliable and valid individual indicators for the Japanese population.

To measure individual efforts toward the SDGs, previous studies have used the official United Nations NY WORLD 2030 survey (United Nations, 2019; Tsunoda, 2020), existing scales as an alternative (Sidiropoulos, 2018), original survey items for each research purpose (Milfont and Sibley, 2012; van der Linden, 2018; Kim et al., 2021; Wang et al., 2022), and cognitive tasks (Hauser et al., 2014; Lange et al., 2018; Langenbach et al., 2019, 2022; Lange and Iwasaki, 2020; Brevers et al., 2021). However, these methods equated pro-environmental commitment with efforts toward SDG and were weak in measuring social and economic efforts (e.g., Lange et al., 2018; Lange and Iwasaki, 2020; Lee et al., 2020; Brevers et al., 2021). In addition, individuals' efforts toward the SDGs were evaluated from a unique perspective, such as interests (van der Linden, 2018; United Nations, 2019; Tsunoda, 2020; Langenbach et al., 2022), attitudes (Wang et al., 2022), values (Kim et al., 2021), behaviors (Hauser et al., 2014; van der Linden, 2018; Langenbach et al., 2019, 2022), etc., and could not be taken into account the diverse approaches of individuals by a single indicator.

Therefore, we established a Japanese version of the Sustainability Consciousness Questionnaire (SCQ). The SCQ measures an individual's awareness and efforts regarding sustainable development (Gericke et al., 2019). The items were created through discussions among experts in biology, pedagogy, and sustainability research, who recognized the coverage of the 15 subthemes of sustainable development and their content validity (Gericke et al., 2019). The SCQ is the first instrument to consider the components of person-level consciousness of sustainability (Gericke et al., 2019), which addressed the problems with the aforementioned indicators. In accordance with UNESCO (UNESCO, 2005; Buckler and Creech, 2014), the SCQ classifies 15 subthemes into three domains: social, economic, and environmental. Thus, compared with previous studies that predominately focused on the environmental domain, the SCQ has diversity in the measurement domain. In addition, the SCQ can measure these subthemes based on three psychological factors: knowledge, attitude, and behavior. In contrast to previous person-level scales, which have been measured from various single psychological perspectives, such as interests, attitudes, values, and behaviors as mentioned above, the SCQ evaluated the diverse consciousness of individuals regarding SDGs.

In particular, this feature of assessing diversity is essential for sustainability research in Japan, as a peculiar discrepancy exists: many Japanese do not know the word “sustainability,” yet Japanese national-level efforts rank high worldwide (Sachs et al., 2021). Some studies have concluded that this discrepancy is due to the Japanese people's cultural personality of valuing the old (Silva et al., 2017; Sirola et al., 2019; Dhir et al., 2021). However, this perspective alone cannot explain Japanese commitment to sustainability except in the environmental domain. To resolve this discrepancy, discussing diverse sustainability efforts in comparison with other cultures is necessary. In this respect, creating a Japanese version of the SCQ, which can measure diverse aspects and be used worldwide (e.g., Berglund et al., 2014, 2020; Gericke et al., 2019; Yuksel and Yildiz, 2019; Marcos-Merino et al., 2020), is significant. However, no scale that can measure individual efforts toward sustainability in Japan exists in the first place.

In this study, we developed a Japanese version of the SCQ and examined its reliability and validity. This research consisted of three studies. In Study 1, we created a Japanese version of the SCQ and confirmed whether the same factor structure as in the original version could be present in the Japanese version. In Study 2, we examined the factor structure and reliability. In Study 3, we investigated whether the constituent elements of the Japanese version of the SCQ appropriately measured relevant psychological characteristics by examining the construct validity of the constituent elements.

## Study 1: Creation of the Japanese version of the sustainability consciousness questionnaire and confirmation of factor structure

**Figure 1:**
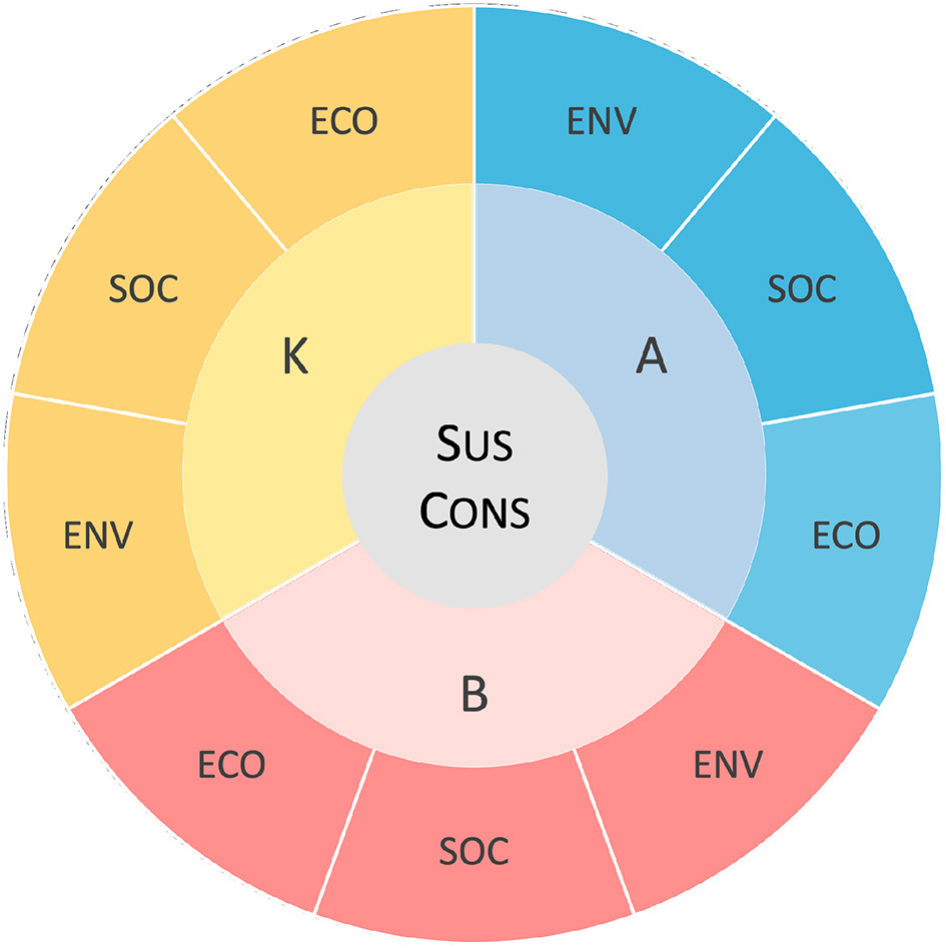
Conceptual representation of the sustainability consciousness questionnaire. K, knowingness; A, attitudes; B, behavior; ECO, economic; SOC, social; ENV, environmental; Sus Cons, sustainability consciousness.

**Figure 2:**
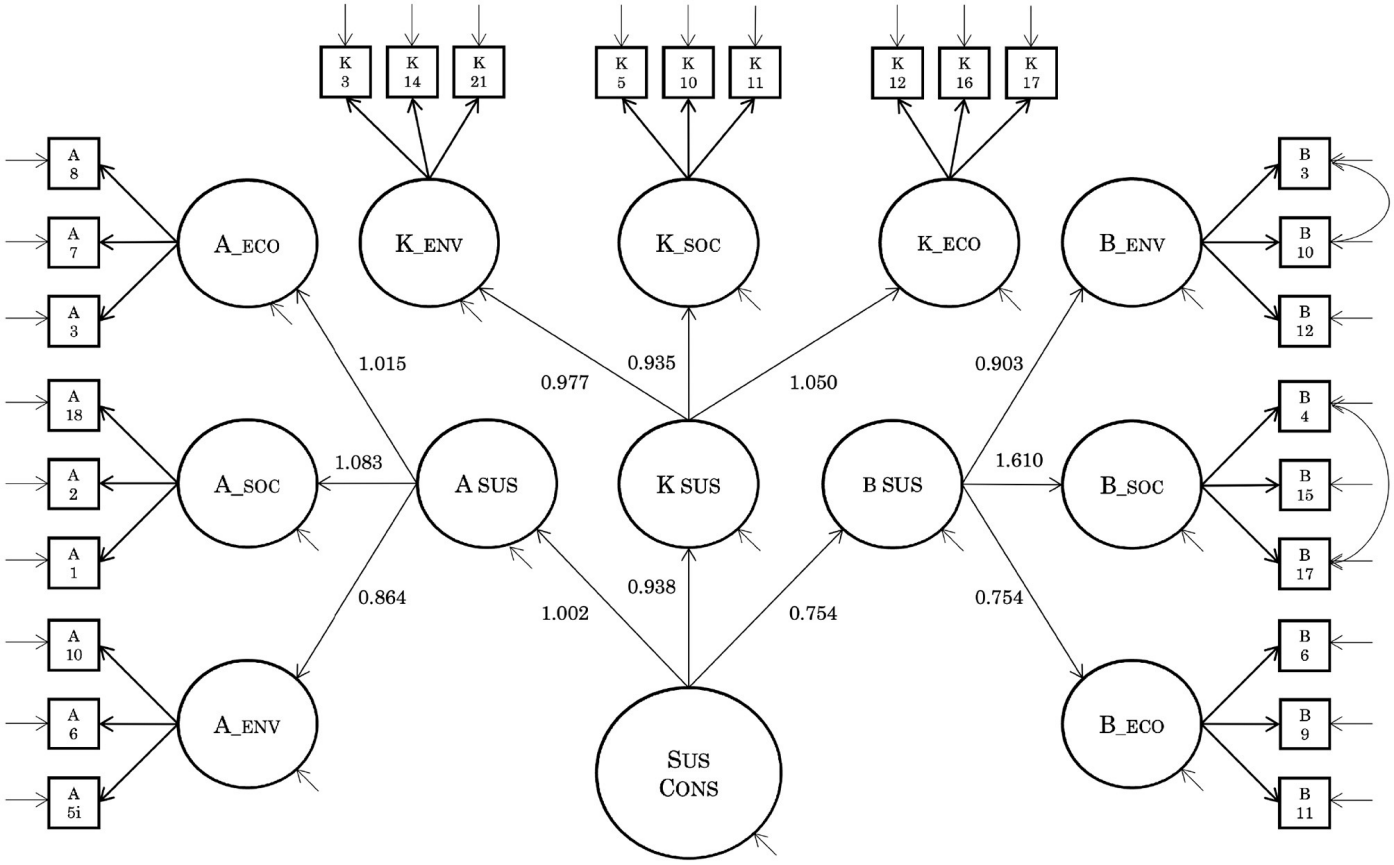
Result of fitting the factor structure of the original version of SCQ to the data in Study 1. The characters in squares (e.g., A5i) indicates the item code in the original study of SCQ (see Supplementary Table 1 for details). Measurement errors are omitted from the notation. When items are connected by an arrow, an error correlation is assumed between them, following the original factor model. K, knowingness; A, attitudes; B, behavior; ECO, economic; SOC, social; ENV, environmental; SUS CONS, sustainability consciousness.

In Study 1, we created a Japanese version of the SCQ and examined factor validity by assuming that the same three-level, nine-factor structure applied to the Japanese version, as in the original version. The SCQ categorizes the 15 subthemes into three domains: social, economic, and environmental, and further measures consciousness toward these subthemes in terms of three psychological factors: knowingness, attitude, and behavior. Here, knowingness is defined as “the state of mind in which a person thinks something to be the case” (Gericke et al., 2019) and measures “what people acknowledge as the necessary features of sustainable development.” Attitude is defined as “an enduring positive or negative feeling about some object, person or issue” (Chaiken and Baldwin, 1981; Kollmuss and Agyeman, 2002) and measures “attitudes toward the sustainable development issues.” Behavior is defined as “the tendency of a respondent to engage in behavior in favor of, or opposed to, the attitude object” (Eagly and Chaiken, 1993) and measures “what people do in relation to the SD issues under consideration.” As a result, the SCQ consists of Layer 1: Sustainability Consciousness; Layer 2: Sustainability Knowingness, Sustainability Attitude, Sustainability Behavior; and Layer 3: (Knowingness, Attitude, Behavior) × (Environment, Social, Ecological aspects) (Figures 1, 2).

The current version of the SCQ consists of the 49-item SCQ-L and its short-version, the 27-item SCQ-S (Gericke et al., 2019). Gericke et al. (2019) noted that SCQ-L and SCQ-S scores are strongly correlated, exhibiting *r*-values of 0.82–0.95. As the SCQ-L requires additional effort to answer, it is recommended that the SCQ-S be used unless valid reasons exist to use the SCL-L (Gericke et al., 2019). As a matter of fact, the Taiwanese (Berglund et al., 2020) and Spanish (Marcos-Merino et al., 2020) versions of the SCQ were developed using the SCQ-S.

## Materials and methods

### Creation of the Japanese version of the SCQ

The Japanese version of the SCQ was created after permission for translation was obtained from the corresponding author of the original study. To comprehensively examine the factor structure, the SCQ-L was translated. The final translation is presented in Supplementary Table 1.

Initially, the first author, who majored in psychology, translated all the items into Japanese. Then, the first author adjusted the expressions of Items 38 and 42, whose contents are not typical in Japanese culture. Item 38 in the original version asked whether food waste should be separated from other types of garbage. However, in Japan, food waste must be disposed of separately as combustible garbage. Therefore, the wording was adjusted to ask whether garbage should be appropriately sorted in general. For item 42, the term “cafeteria committee” was deleted because such a committee is not common in Japan.

Next, the authors conducted discussions and determined the final translations of the items. The Japanese items were back translated into English using an English translation service (Editage, Cactus Communications, Inc.). We checked with the author of the original version whether the semantic contents of these items were the same as the original items. At that time, the original author agreed that there would be no problem changing Items 38 and 42 in the Japanese version. After correcting the Japanese sentences of the items identified as needing correction by the original author and retranslating these items, we obtained confirmation from the original author that the meaning and content of the original version were equivalent.

### Participants and procedures

A cross-sectional survey was conducted using Lancers Inc., a Japanese crowdsourcing service company. After obtaining informed consent, only those who agreed to participate were included in the survey. The sample size was determined according to COSMIN's criteria for excellence (number of items × 7 and ≥ 100, Prinsen et al., 2018), with a requirement of 343 individuals for the 49 items of the SCQ-L. Consequently, 409 persons aged between 19 and 75 years participated in this study. This study was approved by the ethics committee of Kochi University of Technology, Japan (application number: 237).

For the translated version of the SCQ-L with 49 items, participants were asked to what extent the content of each item applied to them, using a 5-point scale (1 = “strongly disagree” to 5 = “strongly agree”). Following the existing SCQ creation method (Berglund et al., 2014), each item was provided with the response option of “don't know”; choices of this option were treated as missing answers. Items were presented in random order for each participant.

### Analysis

We replaced the missing data using the multiple imputation method. Participants who gave many missing responses and the same or random responses were excluded from the analysis (for more information on preprocessing, see Supplementary Methods). Finally, 302 participants (169 males, 129 females, 4 others, mean age = 42.38 ± 9.72 years) were included in the analysis.

After preprocessing, confirmatory factor analysis (MLR method), which assumed the same three-level, nine-factor model as in the original version, was performed. However, this analysis generated an improper solution. Therefore, additional exploratory factor analyses (maximum likelihood method and Oblimin rotation) were performed to estimate the number of factors and their factor items. Based on the results of the exploratory factor analyses, we again performed confirmatory factor analyses, parceling the items into each of the nine factors and assuming error correlations between parcels in the same domain (social, economic, and environmental). An additional confirmatory factor analysis followed the method described by Gericke et al. (2019). First, by comparing the goodness of fit of the analysis assuming, one, two, or three factors, we examined whether the items were explained by more than one factor and which factor structure was appropriate. Next, we examined the hierarchy of the model by calculating and comparing the goodness of fit of the model with the addition of a higher-order factor to explain these factors.

The fit of the confirmatory factor model was determined using CFI, TLI, and RMSEA, as in the development of the original version of the scale. CFI and TLI range from 0 to 1, with higher values indicating better model fit. As an empirical criterion, values of 0.90 or higher are considered to indicate good fit (Bentler and Bonett, 1980). Lower values of RMSEA suggest better fit: empirically, 0.05 or less denotes good fit, 0.05–0.10 denotes moderate fit, and 0.10 or more denotes poor fit (Browne and Cudeck, 1992). Strictly speaking, CFI, TLI, and RMSEA cannot be used to evaluate the hierarchy of the model (see the discussion in the Supplementary Methods). This limitation is addressed in Study 2.

The criterion for significance in statistical tests was *p* < 0.05, two-tailed (Neyman et al., 1933). All analyses were performed using R4.1.2 (R Core Team, 2022). The detailed analysis method is presented in the Supplementary Methods.

## Results and discussion

### Factor structure

Study 1 created a Japanese version of the SCQ and verified its factor structure through a cross-sectional survey. The results of descriptive statistics are presented in Supplementary Table 2.

First, confirmatory factor analysis was performed, assuming the original three-level nine-factor model (Figure 2 and Supplementary Table 3). The goodness of fit was acceptable (RMSEA = 0.06, CFI = 0.848, TLI = 0.828). However, the solution was improper as parameter values exceeded 1.000 (Bollen, 1987) (Figure 2). Therefore, we investigated the correlations between these factors. There was an abnormality in which the correlation coefficient between factors exceeded 1.000, and a relatively strong correlation was observed between the factors as a whole (Supplementary Table 4). That is, there was multicollinearity among the factors, suggesting that the factor structure was not appropriate. In summary, the Japanese of the SCQ version did not support the three-level nine-factor model structure of the original version.

### Re-examination of the factor structure

#### Items and number of factors

**Figure 3:**
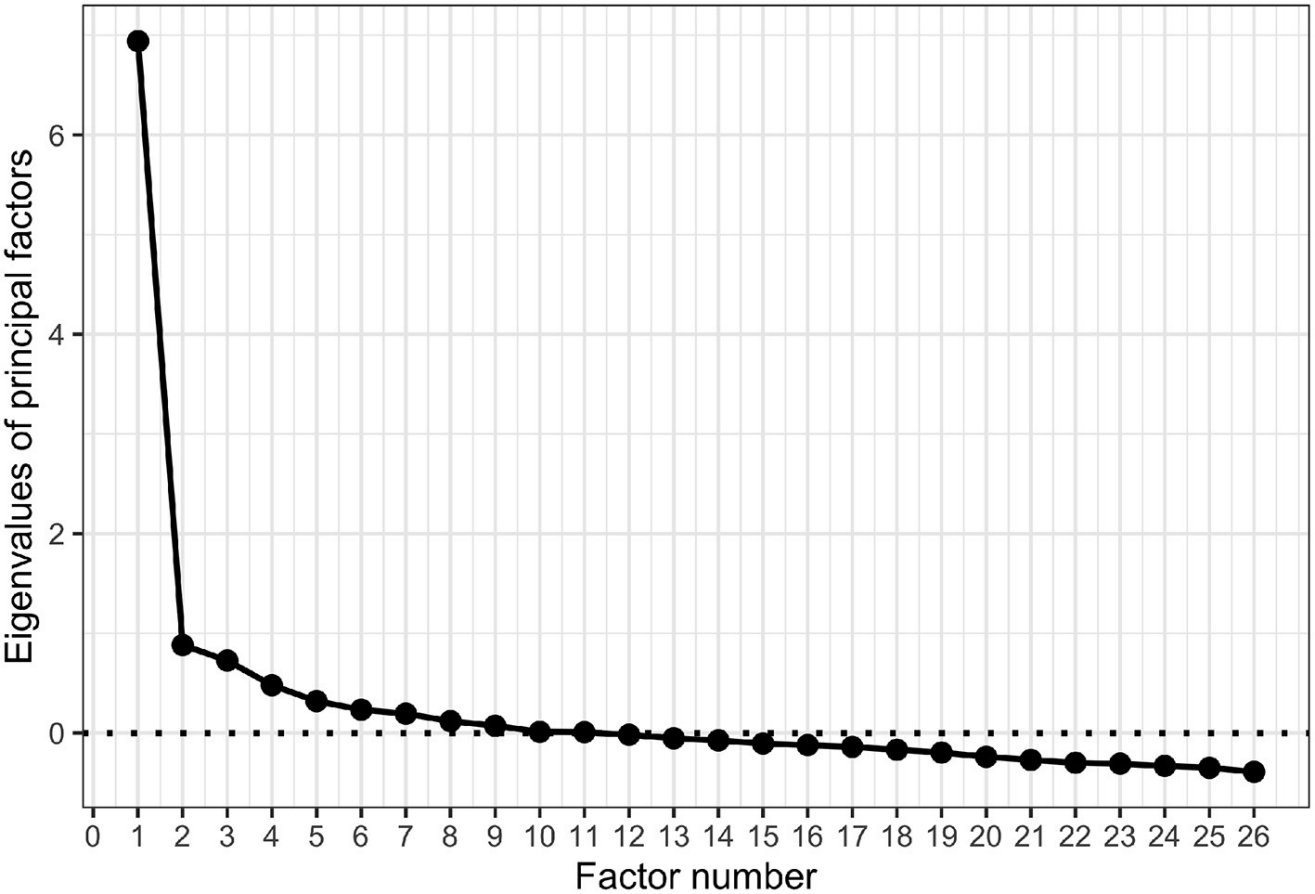
Scree plot of eigenvalues in exploratory factor analysis.

Given the aforementioned results, we re-analyzed the acquired data and re-examined the factor structure. The results of several exploratory factor analyses indicated that it was reasonable to delete items 46 and 47, which had low factor loadings in the Japanese version of the SCQ, and to add item 49, which was included in the same factor in the SCQ-L (Supplementary Results). In addition, it was reasonable that these items were explained by two factors according to Cattell's criterion of the scree plot (Figure 3). Knowingness and Attitude items were regarded as the same factor (Table 1).

**Table 1:** Rotated component matrix for two factor model.

**Item number**	**Item code**	**Factor 1**	**Factor 2**
SCQ_11	K10 (SOC)	0.719	−0.064
SCQ_8	K5 (SOC)	0.677	−0.079
SCQ_28	A18 (SOC)	0.654	−0.061
SCQ_23	A1 (SOC)	0.638	−0.085
SCQ_12	K11 (SOC)	0.623	−0.017
SCQ_15	K12 (ECO)	0.615	0.029
SCQ_30	A7 (ECO)	0.590	0.045
SCQ_17	K17 (ECO)	0.585	0.086
SCQ_40	B4 (SOC)	0.546	−0.109
SCQ_4	K14 (ENV)	0.539	−0.002
SCQ_21	A10 (ENV)	0.525	0.163
SCQ_19	A5i (ENV)	0.482	−0.015
SCQ_16	K16 (ECO)	0.463	0.080
SCQ_29	A3 (ECO)	0.452	0.262
SCQ_38	B10 (ENV)	0.450	0.130
SCQ_45	B17 (SOC)	0.416	0.164
SCQ_6	K21 (ENV)	0.411	0.110
SCQ_31	A8 (ECO)	0.349	0.184
SCQ_24	A2 (SOC)	0.344	0.210
SCQ_1	K3 (ENV)	0.314	0.230
SCQ_20	A6 (ENV)	0.281	0.227
SCQ_39	B12 (ENV)	0.048	0.597
SCQ_44	B15 (SOC)	−0.106	0.541
SCQ_35	B3 (ENV)	0.144	0.524
SCQ_49	B16 (ECO)	−0.043	0.405
SCQ_48	B11 (ECO)	0.042	0.387

The Item Number indicates the item number in the original version of the SCQ. “i” indicates a reversed item. The characters in parentheses, i.e., ENV, SOC, and ECO, indicate the factor to which the item belongs. K, knowingness; A, attitudes; B, behavior; ECO, economic; SOC, social; ENV, environmental.

Thus, in subsequent analyses, it was assumed that the Japanese version of the SCQ consisted of these items and was explained by two factors: knowingness/attitude and behavior. While some items were included in different factors, we did not exchange them among the factors in Study 1, considering the indeterminacy of the factor rotation and interpretability (for detailed reasons, see Supplementary Results). Whether these items had appropriate factor loadings was re-examined in Study 2.

#### Model comparison

We examined the factor structure by performing confirmatory factor analysis and calculating the goodness of fit. Here, we parceled the factor items and assumed correlated errors between the same domain (society, economy, and environment) parcels to examine which models provided suitable solutions.

**Figure 4:**
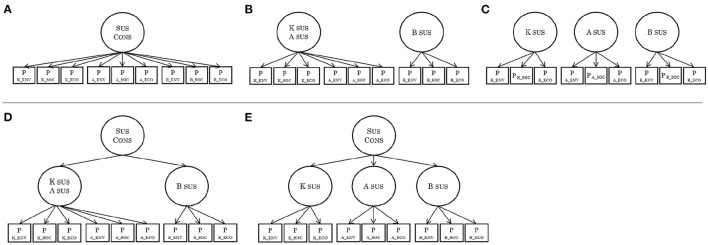
Overview of factor structures. **(A)** One-factor model; **(B)** Two-factor model; **(C)** Three-factor model; **(D)** Two-factor hierarchical model; **(E)** Three-factor hierarchical model. P for each factor item indicates that the corresponding data were parceled. K, knowingness; A, attitudes; B, behavior; ECO, economic; SOC, social; ENV, environmental; Sus Cons, sustainability consciousness.

The results of confirmatory factor analysis assuming a two-factor model (knowingness/attitude, behavior; Figure 4B) are shown in Table 2. The results of a one-factor model (sustainability consciousness; Figure 4A) and a three-factor model (knowingness, attitude, and behavior; Figure 4C) of each layer in the original model are also shown. The three-factor structure fits the data best when only the fitness value was considered. Note that the nine-factor model was excluded from the analysis because the prior confirmatory factor analysis indicated that the correlations between the factors were strong.

**Table 2:** Summary of model fit in Study 1.

**Model**	**RMSEA**	**CFI**	**TLI**
(1) One-factor model	0.102	0.949	0.899
(2) Two-factor model	0.061	0.983	0.963
(3) Three-factor model	0.030	0.996	0.991
(4) Two-factor hierarchical model	0.065	0.982	0.959
(5) Three-factor hierarchical model	0.030	0.996	0.991

There was a slight difference between the results of the three-factor model and the three-factor hierarchical model after the third decimal place.

Next, we examined the hierarchy of the factor structure. A two-layer model (Figures 4D, E) that assumed two or three subfactors of SC fit the data without generating improper solutions. However, compared with the one-layer model, the two-layer model exhibit the same or worse goodness-of-fit. The results of all confirmatory factor analyses are presented in Supplementary Tables 12–16.

These results indicate that the Japanese version had a single-level two-factor structure according to exploratory factor analysis and a single-level three-factor structure according to goodness-of-fit criteria. However, in Study 1, the items were parceled, and this was insufficient to determine whether each scale item appropriately fit the model. Therefore, in Study 2, we examined the factor structure of the Japanese version of the SCQ without parceling and verified its reliability.

## Study 2: Examination of factor structure and reliability of the Japanese version of the sustainability consciousness questionnaire

In Study 2, we examined the validity of the factor structure for the complete set of items in the Japanese version of the SCQ without parceling. Furthermore, by calculating the internal consistency of the constituent factors, we clarified the measurement reliability of the factors.

Specifically, we performed confirmatory factor analyses assuming two-factor and three-factor models as well as one-factor, two-factor hierarchical, and three-factor hierarchical models that did not produce improper solutions in Study 1. We then compared the fit of these models with the data. The hypotheses for this study were as follows: First, we hypothesized that Bayesian information criterion (BIC) would not support a hierarchical structure as in Study 1. Second, we hypothesized that the three-factor model would exhibit the best fit, as in Study 1.

However, as is clear from the scree plot of Study 1 (Figure 3), the greater the number of factors, the more variance in the data tends to be explained. Thus, we did not determine the factor structure of the model using goodness-of-fit measures alone. Indeed, in the exploratory factor analysis of Study 1, the two-factor structure was assumed to be the most appropriate model, and knowingness and attitude items tended to be explained as almost the same factor. Therefore, if the three-factor model exhibited the best fit, then we also examined the correlations among the three factors. In particular, when the correlation between knowingness and attitude factors in the three-factor model was strong, we adopted a single-layer two-factor model, which was estimated as most appropriate as the factor structure of the Japanese version of the SCQ, according to the exploratory factor analysis in Study 1.

## Materials and methods

### Participants and procedures

An online survey was conducted to determine factor structure. We enlisted 361 individuals (18–75 years) who did not participate in Study 1.

The overall procedure was similar to that in Study 1. However, based on the trend in missing responses in Study 1, we modified some procedures. First, in Study 1, there was a tendency for many missing answers to be produced for questions that included the word “sustainable development.” Therefore, we inserted an explanation of sustainable development and checked in advance whether the term was understood before the participants completed the questionnaire. Next, we added the Directed Questions Scale (Maniaci and Rogge, 2014) to the questionnaire, as some participants answered randomly in Study 1. The Directed Questions Scale consists of two items: “Choose strongly disagree for this question” and “Choose neither agree nor disagree for this question”; these were inserted within the scale randomly.

### Analysis

We excluded participants who gave incorrect answers on the Directed Questions Scale (*n* = 11) and performed the same preprocessing as in Study 1 (*n* = 48). As a result, 302 participants (142 males, 158 females, 2 others; mean age = 42.32 ± 11.27 years) were included in the analysis. Confirmatory factor analyses were performed for the models that did not produce improper solutions in Study 1; the models were compared by calculating goodness-of-fit. Items were not parceled. As in Study 1, we assumed that errors were correlated between items in the same domain (that is environment, society, and economy). We also examined the correlations among the factors for factor models judged to have an appropriate fit. Finally, based on classical test theory, the reliability of each factor was examined by calculating the internal consistency using Cronbach's α and McDonald's ω. All analyses were performed using R's Lavaan package (Rosseel, 2012) and psych package (Revelle, 2022).

We used BIC as the main goodness-of-fit index, which can be used for the relative comparison of models, including nested models (Schwarz, 1978). The BIC is an index based on the information criterion; the smaller the value, the better the fit (Raftery, 1996). CFI, TLI, and RMSEA are also shown in the results, following common practice. However, it should be noted that CFI, TLI, and RMSEA have methodological limitations in assessing model hierarchy (see details in the Supplementary Methods).

## Results and discussion

### Model comparison

The results are presented in Table 3. A comparison of the goodness-of-fit of the models showed that the three-factor model and three-factor hierarchical model had the lowest BIC values, indicating that these models fit the data best, as in Study 1. However, it should be noted that there was almost no difference in index values between the three-factor model and two-factor model.

**Table 3:** Summary of model fit in Study 2.

**Model**	**BIC**	**RMSEA**	**CFI**	**TLI**
(1) One-factor model	15,968.080	0.051	0.926	0.879
(2) Two-factor model	15,941.170	0.045	0.941	0.904
(3) Three-factor model	15,935.930	0.043	0.948	0.914
(4) Two-factor hierarchical model	15,946.880	0.046	0.941	0.902
(5) Three-factor hierarchical model	15,935.930	0.043	0.948	0.914

There was a slight difference between the results of the three-factor model and the three-factor hierarchical model after the third decimal place.

### Factor correlations

Given the results of Study 1, there was a high possibility that knowingness and attitude factors could be explained as common factors. Therefore, we examined factor correlations in these models. In both models, the correlation between knowingness and attitude was strong (both *r*s = 0.906), indicating that the knowingness and attitude factors were similar (Supplementary Tables 17, 18).

Next, we examined the factor correlation for the two-factor model. The knowingness/attitude factor and the behavior factor also exhibited a strong correlation (*r* = 0.776). However, given the scree plot in Study 1 and the improved BIC values as compared with the one-factor model, we considered it reasonable to assume that the Japanese version of the SCQ consisted of two factors, considering the interpretability of the scale.

### Reliability

Finally, we examined the reliability of the knowingness/attitude and behavior factors. The knowingness/attitude factor showed sufficient internal consistency (α = 0.889 and ω = 0.902). In contrast, the behavior factor was slightly insufficient (α = 0.688 and ω = 0.772). However, in the original version, α = 0.72, which is comparable to the value for the current Japanese version of the scale. Considering that the behavior factor reflects efforts in various domains such as the environment, society, and economy, this internal consistency value was considered to be acceptable.

**Figure 5:**
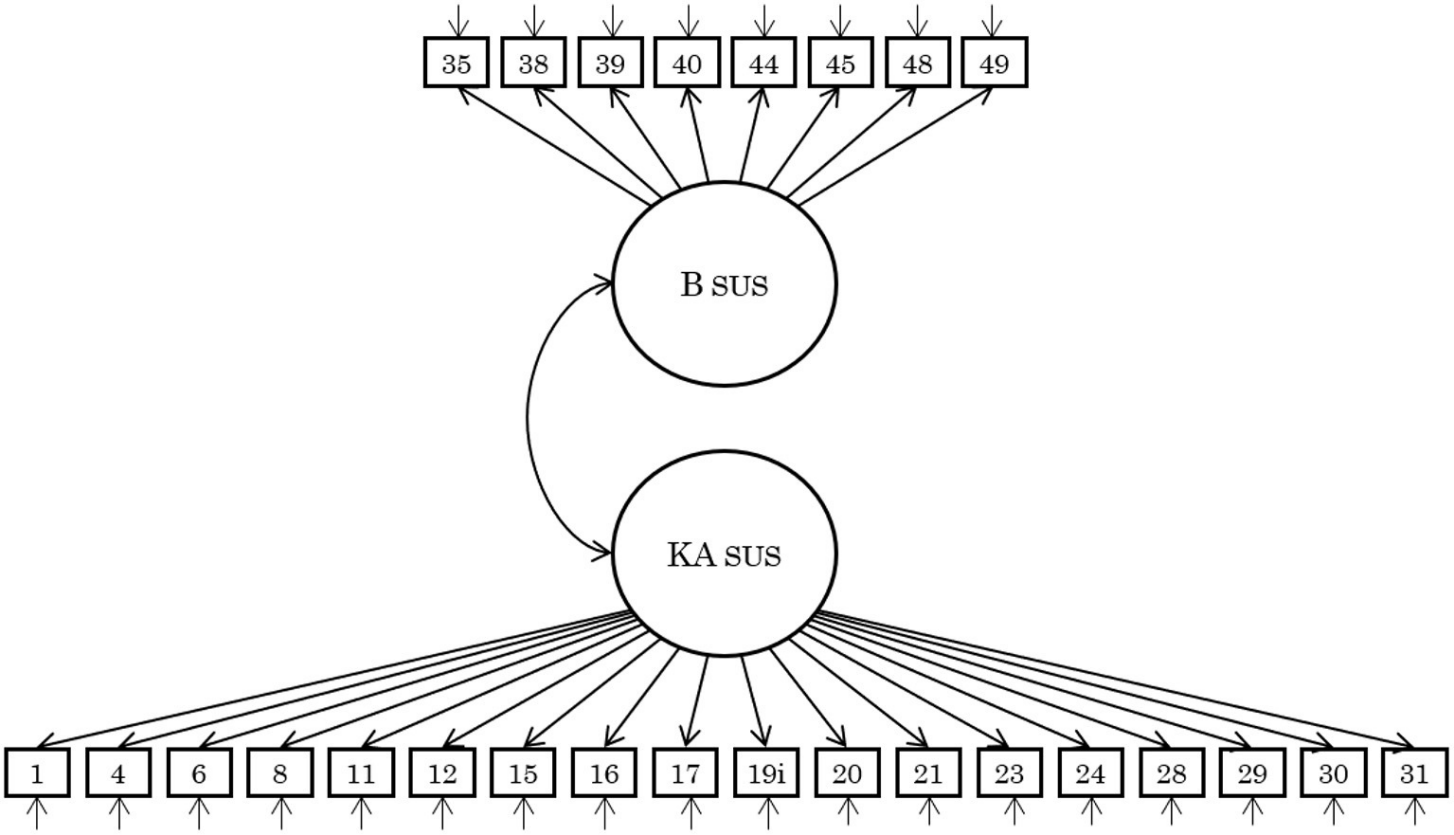
A model of the Japanese version of sustainability consciousness questionnaire. The number in squares indicates the item number of the SCQ (see Supplementary Table 1 for details). Measurement errors are omitted from the notation. Correlated errors were assumed with each domain (i.e., economics, social, environmental). KA, knowingness/attitude; B, behavior.

Therefore, given the model's fit to the data, interpretability, and reliability, it is reasonable to propose that the Japanese version of the SCQ is a single-level two-factor model consisting of knowingness/attitude and behavior. The confirmatory factor analysis results for the two-factor model (Figure 5) are shown in Table 4 and the results of all other confirmatory factor analyses are shown in Supplementary Tables 19–22.

**Table 4:** Factor loading of each item for the two-factor model.

**Item**	**Factor loading**	***SE***	***Mean***	***SD***
**Knowingness/attitude (**α **= 0.889**, ω **= 0.902)**				
SCQ_1	0.472	0.054	3.806	0.760
SCQ_4	0.510	0.053	4.189	0.643
SCQ_6	0.457	0.065	4.199	0.605
SCQ_8	0.590	0.049	4.169	0.676
SCQ_11	0.637	0.047	4.178	0.646
SCQ_12	0.585	0.056	4.162	0.650
SCQ_15	0.649	0.045	4.182	0.562
SCQ_16	0.527	0.047	3.742	0.715
SCQ_17	0.629	0.048	4.050	0.711
SCQ_19i	0.415	0.060	1.954	0.838
SCQ_20	0.419	0.059	3.669	0.837
SCQ_21	0.603	0.040	4.332	0.646
SCQ_23	0.535	0.058	4.275	0.600
SCQ_24	0.520	0.050	3.871	0.807
SCQ_28	0.570	0.045	4.355	0.609
SCQ_29	0.640	0.046	4.093	0.676
SCQ_30	0.605	0.049	4.342	0.589
SCQ_31	0.482	0.055	3.493	0.818
**Behavior (**α **= 0.688**, ω **= 0.772)**				
SCQ_35	0.566	0.062	3.854	0.733
SCQ_38	0.520	0.067	4.286	0.656
SCQ_39	0.533	0.066	3.536	0.983
SCQ_40	0.439	0.071	3.886	0.763
SCQ_44	0.395	0.081	2.539	0.999
SCQ_45	0.579	0.051	4.182	0.613
SCQ_48	0.388	0.058	3.377	0.884
SCQ_49	0.288	0.076	3.517	1.052

*SE*, Standard Error; *Mean*, Mean item score; *SD*, Standard Deviation of the item score. i indicates that the item is reversed.

## Study 3: Examination of the construct validity of the Japanese version of the sustainability consciousness questionnaire

The Japanese version of the SCQ was constructed based on two factors: knowingness/attitude and behavior. However, we did not examine whether the psychological characteristics assumed by these factors could be appropriately measured. Therefore, in Study 3, we examined the construct validity of the Japanese version of the SCQ.

To examine the construct validity, we focused on van der Linden (2015) climate change risk perception model (CCRPM) as an external criterion. CCRPM is a psychosocial model that comprehensively explains perceived climate change risk. The components were defined based on a comprehensive review of previous research on climate change risk perception (van der Linden, 2015), which consisted of gender, political party, knowledge of the causes, impacts, and responses to climate change, social norms, value orientations, affect, and personal experience with extreme weather. In the CCRPM, the similarities and differences among the constituent elements were well-examined, and the survey items were prepared with sufficient consideration given to the heterogeneity among the components. A large sample of UK national (van der Linden, 2015) and nationwide (van Eck et al., 2020) studies have revealed that approximately 70% of the variance in climate change risk perception can be explained by this survey; this result has been replicated in multiple similar studies (Geiger et al., 2018; Xie et al., 2019; Han et al., 2022).

Therefore, we clarified the construct validity of the SCQ by showing that its factors were appropriately correlated with the extracted CCRPM scales for measuring knowingness, attitude, and behavior. Specifically, we extracted knowledge and holistic affect scales from the CCRPM to measure SCQ's knowingness and attitude factors (see Methods for details). Furthermore, we extracted the pro-environmental behavior scale (van der Linden, 2018), whose relationship with CCRPM has been investigated to measure SCQ's behavior factor. By showing that these scales have uniquely weak to moderate correlations (*r* = 0.30–0.50) with knowingness/attitude and behavior factors, we demonstrated construct validity.

We also examined the correlation between total SCQ scores and measures related to sustainability. First, we assumed that the SCQ would have a weak positive correlation with generativity maintaining (*r* = 0.2–0.4). Here, generativity maintaining represents concern and transmission to the next generation and we considered it close to the SDGs' core definition: “a development that meets the needs of the present generation without compromising the ability of future generations to meet their own needs” (Al-Athel et al., 1987). In addition, we assumed that the SCQ would have a weak positive correlation with empathy (*r* = 0.2–0.4) and a weak negative correlation with social dominance orientation (*r* = −0.2 to −0.4). Several studies have shown that empathy is involved in pro-environmental attitudes and behaviors (Berenguer, 2007, 2010; Matewos et al., 2022), and empathy may also be involved in sustainability (Brown et al., 2019). In contrast, social dominance orientation, which tends to stratify society according to power and status, has a negative correlation (*r* = −0.30) with pro-environmental behavior (Stanley and Wilson, 2019). Finally, we clarified the discriminant validity of the SCQ by examining its relationship with the Big Five personality traits. Several studies have investigated the relationship between sustainability and the Big Five personality traits; overall, a trend has been reported that relates sustainability attitudes to agreeableness and openness to experience (e.g., Hirsh, 2010, 2014; Milfont and Sibley, 2012). Therefore, by showing that SCQ had a weak positive correlation only with agreeableness and openness to experience among the Big Five personality traits (*r* = 0.2–0.4), we planned to clarify the discriminant validity of the SCQ.

## Materials and methods

### Participants and procedures

An online survey was again conducted. Participants were recruited from Lancers Co., Ltd., and asked to complete the SCQ and seven other scales. As in Study 2, participants read an explanation of sustainable development before answering the questionnaire. The questionnaire consisted of three blocks: (1) the Japanese version of the SCQ, (2) three scales extracted from the CCRPM to examine construct validity, and (3) four other scales for measuring generativity, empathic concern, social dominance orientation, and Big Five personality traits. The questionnaire included Directed Questions Scale items, similar to Study 2. The order of the scales within each block was randomized for each participant. Furthermore, the order of the items for all scales was randomized for each participant.

We recruited individuals who did not participate in Study 1 and 2; 456 individuals (aged 19–69 years), a similar number to those who participated in Study 1 and 2, participated in Study 3. Assuming a correlation coefficient of 0.2, a one-sided test with a significance level of 5%, and a power of 80%, the required number of participants was 153.

### Measures

#### Scales for construct validity

To examine the validity of the construct, participants responded to the knowledge about climate change and the holistic affect scale of the CCRPM (van der Linden, 2015) as measures related to knowingness and attitude factors of the SCQ. We also asked participants to complete the pro-environmental behavior scale developed by van der Linden (2018), whose correlation with the CCRPM scale has been investigated as a measure related to the behavior factor of the SCQ. All scales were translated into Japanese.

##### Knowledge about climate change

A 13-item scale was used that probes knowledge of the impacts and consequences of climate change. van der Linden (2015) noted that there is an essential difference between “subjective knowledge” (i.e., what most people believe to be confirmed) and “objective, evidence-based knowledge” (e.g., facts with scientific consensus, such as that burning fossil fuels effects climate change), and developed a scale to measure the degree of knowledge of the latter. The scale requires participants to choose from three options for each item. For example, regarding global sea level, the choices are that it will increase, decrease, or not change due to climate change. For each item, the option “Don't know” was provided. Answers to the option were treated as incorrect, eliminating the risk of respondents giving the correct answer by mistake due to random responses. Of the 13 items listed, seven were affected by climate change, and six were not. Responses were converted into a binary score for correct (1) or incorrect (0). The correct responses to all items are based on scientific evidence and can be judged objectively by referring to expert reports, such as the IPCC5. The scale score was the sum of the number of correct answers.

##### Holistic affect regarding climate change

A three-item scale was used that asks how one feels about climate change. For the question “I feel that climate change is…,” we asked the participants to answer three adjective scales on a 7-point scale: 1 = very unpleasant, 7 = very pleasant; 1 = very unfavorable, 7 = very favorable; and 1 = very negative, 7 = very positive. Thus, higher scores indicate a more positive evaluation of climate change, whereas lower scores indicate a more negative evaluation. The CCRPM states, “if affect is operationalized as an evaluative measure (like/dislike)–this tends to be conceptually closer to a measure of attitude.” The CCRPM also defines “attitude” as “the affect for or against a psychological object”; this definition is very similar to the SCQ definition of attitude (see Section 3). Therefore, this study used holistic affect as a scale to examine the construct validity of attitudes.

##### Pro-environmental behavior

This behavior was assessed *via* a 21-item scale that asks how often pro-environmental behaviors have been executed in the past 4 weeks. Examples include “Turned off your car when idle for longer than 30 s (except in traffic),” “Purchased a more fuel-efficient automobile,” and “Bought locally grown and produced food.” Participants were asked to respond on seven-point scale: 1 = “never” to 7 = “very frequently.” Some items could not be answered unless the respondent owned a car or home appliance (e.g., “Turned off your car when idle for longer than 30 s (except in traffic”); thus, a “Not applicable” option was added to such items, following (United Nations, 2019). In addition, some actions could not be executed often, such as “Purchased a more fuel-efficient automobile.” For such items, we asked participants to answer 7 (“very frequently”) if they had executed the actions, and 1 (“never”) if they had not executed the actions, also following (van der Linden, 2018). The scale score was taken as the mean item score, excluding items that received “Not applicable” responses.

#### Generativity maintaining

We used the generativity maintaining factor of the Japanese version of the Loyola Generativity Scale (Marushima and Arimitsu, 2007; original version, McAdams and de St. Aubin, 1992). The generativity maintaining factor consists of five items, such as “I try to pass along the knowledge I have gained through my experiences.” Responses are collected using a four-point scale, ranging from 1 = “not at all applicable” to 4 = “very applicable.”

#### Empathic concern

The empathic concern factor of the Japanese version of the Interpersonal Reactivity Index (Himichi et al., 2017; original version, Davis, 1980) was used to measure empathy for others. The scale consists of seven items, and we requested responses on a five-point scale ranging from 1 = “does not describe me well” to 5 = “describes me very well”.

#### Social dominance orientation

The Japanese version of the SDO_6_ scale (Mifune and Yokota, 2018; original version, Pratto et al., 1994) was used to measure social dominance orientation. The SDO_6_ scale consists of 16 items, each of which is rated on a seven-point scale ranging from 1 = “extremely oppose” to 7 = “extremely favor.

#### Big five personality

The Big Five personality traits were measured using the Japanese version of the Ten-Item Personality Inventory (Oshio et al., 2012; original version, Gosling et al., 2003). The Ten-Item Personality Inventory consists of five personality factors: Extraversion, Agreeableness, Neuroticism, and Openness to experience. Participants were asked to provide responses on a seven-point scale (1 = “strongly disagree” to 7 = “strongly agree”).

### Analysis

We excluded the data of persons who gave incorrect answers to the Directed Questions Scale (*n* = 15) and performed the same preprocessing as in Studies 1 and 2 (*n* = 68). However, in Study 3, participants who were missing 25% or more of the nine factors were not excluded from the analysis, but their total score of the factor to which the factor item belonged (i.e., knowingness/attitude and behavior) were calculated as missing score. In an outlier test based on Mahalanobis distance, the degree of freedom was the number of questionnaire items, excluding the Directed Questions Scale items. Accordingly, 441 participants (231 males, 207 females, 3 others; mean age = 40.38 ± 10.62 years) were included in the analysis.

To examine the construct validity of the SCQ, Pearson's correlation coefficients were calculated between the knowingness/attitude factor of the SCQ and knowledge about climate change and holistic affect of CCRPM, and the behavior factor of the SCQ and pro-environmental behavior. To examine the validity of the SCQ, Pearson's correlation coefficients were calculated between the total score of the SCQ and the other scales.

## Results and discussion

### Descriptive statistics

Table 5 shows descriptive statistics for each scale. For all scales, kurtosis and skewness were at acceptable levels (i.e., between −2 and +2, George and Mallery, 2010), indicating that scores generally followed a normal distribution.

**Table 5:** Descriptive statistics for the scales in Study 3.

**Measure**	***Mean***	***SD***	***Skewness***	***Kurtosis***
**Sustainability consciousness questionnaire**				
Total	101.460	9.810	−0.430	0.290
Sustainability knowingness/attitude	72.580	7.370	−0.500	0.410
Sustainability behavior	28.870	3.800	−0.220	−0.210
**Climate change risk perception model**				
Knowledge about climate change	6.760	1.810	−0.070	0.820
Holistic affect	6.970	2.660	0.180	−0.890
Pro-environmental behavior	3.080	0.920	0.330	0.280
**Interpersonal reactivity index**				
Empathic concern	24.550	4.090	−0.560	0.330
**SDO**_6_ **scale**				
Social dominance orientation	53.650	10.840	−0.270	0.320
**Ten items personality inventory**				
Extraversion	6.550	2.490	0.240	−0.210
Agreeableness	9.460	2.100	−0.390	0.010
Conscientiousness	7.330	2.490	−0.030	−0.450
Neuroticism	8.890	2.510	−0.180	−0.210
Openness to experience	7.730	2.500	−0.020	−0.420
**Loyola generativity scale**				
Generativity maintaining	10.410	3.010	−0.020	−0.620

### Validity

**Figure 6:**
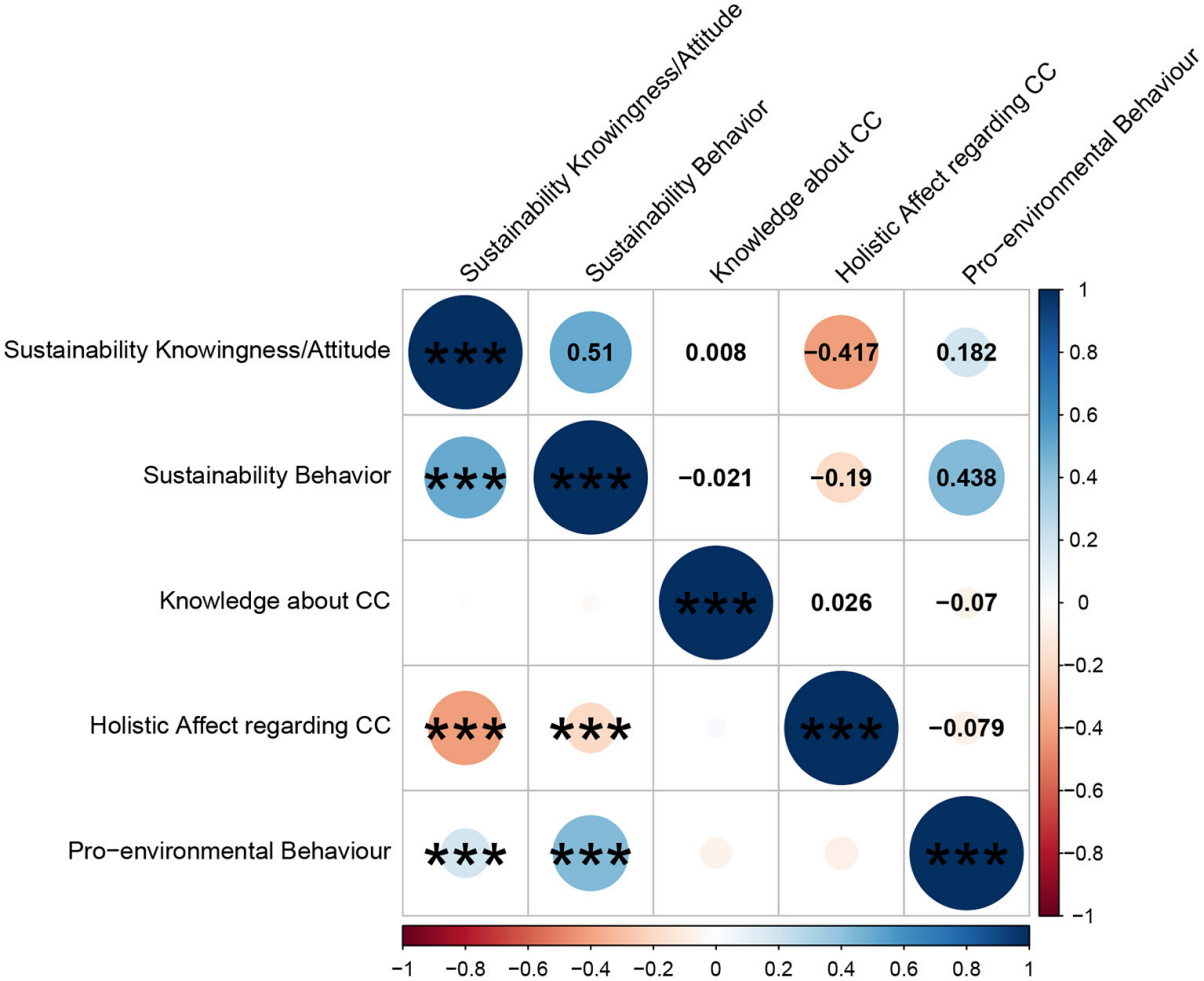
Correlation matrix between the total score of the sustainability consciousness questionnaire (SCQ) and Climate Change Risk Perception Model scales. Correlation coefficients are shown in the upper triangular matrix and significance values are shown in the lower triangular matrix. The color spectrum indicates the magnitude of the correlation coefficient. For holistic affect regarding climate change, higher scores indicate a more positive evaluation of climate change, while lower scores indicate a more negative evaluation. ***p < 0.001.

Next, we examined construct validity. Figure 6 shows Pearson's correlations between the SCQ subscales (knowingness/attitude, behavior) and the CCRPM subscales (knowledge about climate change, holistic affect, and pro-environmental behavior). As expected, SCQ's knowingness/attitude and holistic affect, and SCQ's behavior and pro-environmental behavior exhibited a significant small-to-moderate correlation (*r* = −0.417, *p* < 0.001; *r* = 0.438, *p* < 0.001). However, there was no significant correlation between SCQ knowingness/attitude and knowledge about climate change, contrary to our hypothesis (*r* = 0.008, *p* = 0.880). Therefore, the hypothesis regarding the correlation between the SCQ and CCRPM was only partially supported. However, given the appropriate correlation between SCQ knowingness/attitude and holistic affect, and between SCQ behavior and pro-environmental behavior, the SCQ appeared to have some degree of construct validity.

**Figure 7:**
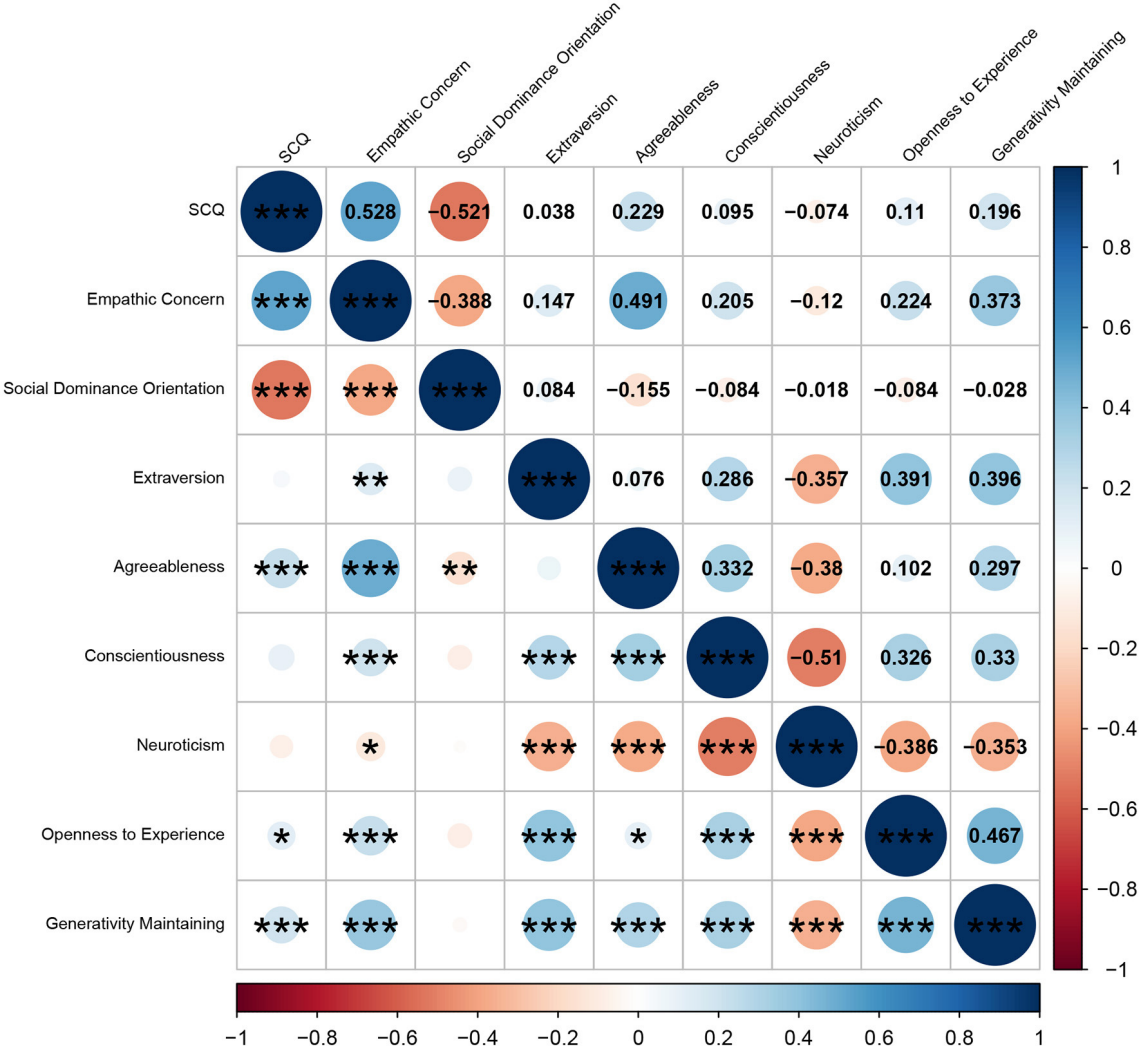
Correlation matrix between the total score of the sustainability consciousness questionnaire (SCQ) and Climate Change Risk Perception Model scales. Correlation coefficients are shown in the upper triangular matrix and significance values are shown in the lower triangular matrix. The color spectrum indicates the magnitude of the correlation coefficient. For holistic affect regarding climate change, higher scores indicate a more positive evaluation of climate change, while lower scores indicate a more negative evaluation. ***p < 0.001, **p < 0.01, *p < 0.05.

Furthermore, we examined the practical utility of the SCQ. Pearson's correlations were examined between the SCQ total score and empathic concern, social dominance orientation, Big Five personality traits, and generativity maintenance. The results are shown in Figure 7. As hypothesized, there was a significant correlation between the SCQ total score and generativity maintenance *r* = 0.196, *p* < 0.001), reflecting interest in future generations aligned with the SDG definition, although the effect size was slightly weaker than expected (*r* = 0.2–0.4). In addition, SCQ scores exhibited moderate correlations with empathic concern (*r* = −0.521, *p* < 0.001) and social dominance orientation (*r* = 0.528, *p* < 0.001), both which have been reported as related to sustainability in many previous studies. Finally, consistent with previous findings (e.g., Hirsh, 2010, 2014; Milfont and Sibley, 2012), SCQ scores were significantly correlated only with the Big Five personality traits of agreeableness (*r* = 0.229, *p* < 0.001) and openness to experience (*r* = 0.110, *p* = 0.034), indicating appropriate discriminant validity.

These results indicated that the SCQ had generally appropriate correlations with psychological indicators associated with sustainability in previous studies; the criterion-related validity of the SCQ was thus supported.

## General discussion

The purpose of this study was to create a Japanese version of the SCQ and verify its reliability and validity. In Studies 1 and 2, we created a Japanese version that is equivalent in content to the original version using a retranslation method and examined its factor structure. The Japanese version was composed of two factors, knowingness/attitude and behavior, and sufficient reliability was shown for these two factors. In Study 3, the Japanese version of the SCQ showed hypothesized correlations with the assumed psychological scales, indicating construct validity. The above results clarify that the Japanese version of the SCQ created in this study is a reliable and valid scale.

In Japan, when considering individual efforts toward SDGs, no prior indicator considered the diversity of the SDGs. However, with development of this questionnaire, which covers 15 subthemes of sustainable development (UNESCO, 2005; Buckler and Creech, 2014), it is now possible to measure individuals' awareness of and commitment to the SDGs in the social, economic, and environmental domains. Furthermore, it was shown that the SCQ items appropriately measure the psychological characteristics that underlie sustainability. This is evident from the SCQ constructs, knowingness/attitude, and behavior, which exhibited appropriate correlations with psychological scales, such as those assessing holistic affect regarding climate change and pro-environmental behavior (van der Linden, 2015, 2018). Further, the SCQ total score exhibited appropriate correlations with psychological characteristics, such as empathy and social dominance orientation, which have been theoretically associated with sustainability (Berenguer, 2007, 2010; Brown et al., 2019; Stanley and Wilson, 2019; Matewos et al., 2022).

In contrast, after careful examination of three studies, it became clear that the Japanese version does not support the hierarchical factor structure assumed by the original version. Indeed, the confirmatory factor analysis of Study 1, which considered the original model structure, generated improper solutions, as indicated by the parameter estimates. Additionally, high correlations were observed among the nine sub-factors. However, these strong correlations are comprehensible, given the structural equation of the original model. In the original hierarchical factor structure that we initially assessed, we were unable to consider the commonality of the environmental, social, and economic domains. For example, in the original model, K_SOC, A_SOC, and B_SOC were considered almost irrelevant factors, with only the sustainability consciousness factor common across all items (see Figure 2). Nevertheless, research on the components of the SDGs has revealed considerable commonality in the individual interests related to SDG items in the environmental, social, and economic domains (Bain et al., 2019). Accordingly, K_SOC, A_SOC, and B_SOC may have a social commonality that was not assumed in the original model; if so, this commonality could increase the factor correlations.

To account for commonalities among the environmental, social, and economic domains, it may be appropriate to assume a single hierarchical model. Indeed, we confirmed that appropriate fit could be obtained in Study 2, by assuming correlated errors among items in the same domain of society, economy, and environment. This idea has been implemented for some items in the original version, and it has been reported that appropriate fit was obtained by assuming such correlated errors (for details, see the SCQ-L creation process in Gericke et al., 2019). Accordingly, we consider that the Japanese version of the SCQ has a single hierarchical structure.

However, it should be noted that the Japanese version of the SCQ consists of two factors: knowingness/attitude and behavior. Thus, the original version's factor structure of knowingness, attitude, and behavior was not supported in the Japanese version. Regarding the items, knowingness and attitude have clearly different content, and it seems contradictory that they are the same factor. This can be understood as consistent by considering the following two possibilities. First, although knowingness and attitude items measure different psychological traits, responses to the items may be similar. In fact, previous research on pro-environmental behavior and climate change has found a clear causal relationship between knowledge and attitudes, whereby knowledge shapes attitudes (Shi et al., 2016; Geiger et al., 2018; Liu et al., 2020; Azizah, 2022; Wong-Parodi and Berlin Rubin, 2022). It is also conceivable that these two characteristics have much in common. In considering this possibility, it should be noted that SCQ's knowingness is a concept that differs from knowledge. Gericke et al. (2019) claim that knowledge about sustainability is not always accurate; it is formulated in a context-dependent manner, such as due to differences in the groups to which one belongs. Therefore, SCQ knowingness is closer to belief than to accurate knowledge (Gericke et al., 2019). Even in this study, there was no correlation with knowledge about climate change of the CCRPM (van der Linden, 2015), which probes scientific knowledge about the causes of climate change. It is possible that the nature of the knowingness factor was consequently regarded as identical to the attitude factor.

## Limitation

There are limitations to how this study examined construct validity. First, to accurately discriminate and understand the concepts of knowingness, attitude, and behavior, we used van der Linden (2015) survey items instead of a questionnaire scale whose reliability and validity had been established. However, the results of van der Linden (2015) have been reproduced in many studies (Xie et al., 2019; Elshirbiny and Abrahamse, 2020; van Eck et al., 2020; Han et al., 2022); hence, it is also possible to be seen the reliability and validity have been sufficiently verified, even if no statistical indicators have been explicitly calculated. In addition, the van der Linden (2015) scale is specific to climate change in the environmental domain. However, to the best of our knowledge, no scales other than that of van der Linden (2015) adequately discriminate among similarities in knowledge, attitudes, and behaviors in the environmental, economic, or social domains. Indeed, construct validity has not been examined in any version of the SCQ (e.g., Michalos et al., 2012; Berglund et al., 2014, 2020; Olsson and Gericke, 2016; Gericke et al., 2019; Yuksel and Yildiz, 2019; Marcos-Merino et al., 2020). This problem could be resolved to a certain extent by developing scales that can appropriately discriminate and measure knowingness, attitude, and behaviors, especially in the economic and social domains.

Additionally, the Japanese version of the SCQ adopted only the core single-layer structure of the original version: knowingness, attitude, and behavior. That the original factor model was not validated in the Japanese version may be due to cultural differences. However, more importantly, there were significant differences in the demographics (age groups) involved in the development of the Japanese and original versions of the SCQ: adults in general in the former and 18-year-olds in the latter. In this study, we assumed the possibility that knowledge acquisition precedes attitudes toward sustainability because the Japanese version's knowingness and attitude factors were identical, despite sufficient content differences. That is, we suggested the possibility that the knowingness and attitude factors may be separate before the beginning of exposure to information about sustainability. Therefore, to further examine the validity of the factor structure, it will be necessary to conduct further studies. First, it is necessary to conduct a longitudinal study including younger age groups. Such a study would likely confirm that measurement invariance is maintained even when demographics differ, that each factor has appropriate responsiveness, and that the measurement error within a subject is sufficiently small. In addition, international comparisons of the factor structures should be conducted.

## Conclusion

Using the Japanese version of the SCQ, awareness and commitment to sustainability can be assessed using two constructs, knowingness/attitude and behavior, considering the 15 subthemes of sustainable development. In particular, these constructs are reliable and valid in the Japanese version of the SCQ. This study examined the construct validity of the SCQ. Considering that the examination of construct validity has been overlooked for any version of the SCQ, the present findings make an important contribution to the literature beyond the creation of the Japanese version of the instrument. In future studies, the Japanese version of the SCQ may help clarify the differences in the structure of people's sustainability consciousness in detail, which may help promote SDG initiatives not only in Japan, but also globally.

## Data availability statement

The datasets presented in this study can be found in online repositories. The names of the repository/repositories and accession number(s) can be found below: https://github.com/HOgishima/The-Japanese-Version-of-the-Sustainability-Consciousness-Questionnaire.

## Ethics statement

The studies involving human participants were reviewed and approved by Kochi University of Technology. Written informed consent for participation was not required for this study in accordance with the national legislation and the institutional requirements.

## Author contributions

HO, AI, SK, and TH contributed to the conception and design of the study. HO organized the database, performed the statistical analysis, and wrote the first draft of the manuscript. TH collected the data and supervised the study. All authors contributed to the manuscript revision, read, and approved the submitted version.

## Footnotes

^1^It consists of the following 15 subthemes: the social subthemes are (i) human rights, (ii) peace and human security, (iii) gender equality, (iv) cultural diversity and intercultural understanding, (v) health, (vi) HIV/AIDS, and (vii) governance; the environmental subthemes are (i) natural resources (water, energy, agriculture and biodiversity), (ii) climate change, (iii) rural development, (iv) sustainable urbanization, and (v) disaster prevention and mitigation; the economic subthemes are (i) poverty reduction, (ii) corporate responsibility and accountability, and (iii) market economy (UNESCO, 2005; Buckler and Creech, 2014).
