# Supplementary material for: Validity and reliability of the Japanese version of the sustainability consciousness questionnaire
Source: Front Psychol. 2023 Mar 15;14:1130550. doi: 10.3389/fpsyg.2023.1130550 (PMC10050387; doi:10.3389/fpsyg.2023.1130550)
Supplement: Supplementary file 1 [file Data_Sheet_1.pdf]

Supplementary Material

1 SUPPLEMENTARY METHODS

1.1 Preprocessing of Data

It is often a problem that the data collected in online surveys contain insufficient effort expended by participants when making responses, which distorts data quality (Takahashi et al., 2022). Therefore, in this study, we performed data cleaning before analysis.

First, according to the conventional SCQ preparation procedure (Berglund et al., 2020), participants with 25% or more missing items for any of the nine factors were excluded from the subsequent analyses. However, for factors consisting of four or fewer items, participants with two or more missing items were excluded.

Next, we imputed missing data because the data were not regarded as missing completely at random (MCAR). When the data are MCAR, excluding participants with missing responses using listwise or pairwise methods results in considerable distortion of the results (Little, 2019). Therefore, we used the multiple imputation method to replace the missing values, using the R package mice (Buuren, 2018).

Finally, to exclude participants who provided singular or random answers, we vectorized the item scores for each participant and calculated the Mahalabinos distance from the overall average (Ghorbani, 2019). Mahalbinos distance has been shown to follow a chi-square distribution, with the number of items as the degrees of freedom (Brereton, 2015). Therefore, we excluded participants for whom the Mahalabinos distance was considered to be dissociated from the mean with $p < .0.01$ probability, given a chi-square distribution with 49 degrees of freedom (Tabachnick and Fidell, 2013).

1.2 Confirmatory Factor Analysis

We assumed a three-level nine-factor model, similar to the original model, and conducted confirmatory factor analysis using structural equation modeling with the Lavaan package of R. Before the analysis, we first checked whether the individual item scores were normally distributed by calculating skewness and kurtosis. Because not all items were normally distributed (Supplementary Table 2), the model was estimated using a robust estimation method (MLR, Yuan and Bentler, 2000), which can be applied to non-normally distributed data, to minimize the risk of improper solutions.

1.3 Exploratory Factor Analysis

Exploratory factor analysis was performed using structural equation modeling. Assuming correlations between factors, oblique factor rotation was performed using oblimin rotation and the maximum-likelihood method. The number of factors was determined based on a scree plot. R's Psych package was used for this analysis.

1.4 Additionally Confirmatory Factor Analysis

Confirmatory factor analysis, assuming a three-level nine-factor factor structure, produced improper solutions. Therefore, after taking countermeasures, confirmatory factor analysis was conducted again in this study.

First, we assumed that the original three-level model produced improper solutions because of local solutions (such as items within the same factor with a locally strong correlation). We parceled the factor items to examine the fit of the factor structure to the data. Parceling is a method of reducing the number of items by adding two or more items (Little et al., 2002). If the model fits the data, parceling may reduce the

goodness of fit, whereas parceling may stabilize the factor estimation if local solutions are found (Little et al., 2002; Baer et al., 2008; Rocha and Chelladurai, 2012). Therefore, parceling was performed on the items included in each of the nine factors of the original version.

In addition, we took measures to address strong factor correlations. We assumed that items belonging to the same domain (society, economy, and environment) may have commonalities, even if they are included in different factors (Gerbing and Anderson, 1984). Therefore, we assumed correlated errors would exist among items parceled in the same domain (society, economy, and environment).

1.5 RMSEA, CFI, and TLI

It should be noted that fit indices such as RMSEA, CFI, and TLI cannot be used to evaluate the validity of hierarchical structures (Marsh et al., 2004; Savalei et al., 2021). RMSEA, CFI, and TLI rate more complex models with many free parameters. On the other hand, a hierarchical factor model is considered a nested single-layer factor model. That is a single-layer factor model adds constraints to the parameters of the hierarchical factor model to limit the number of free parameters (Raykov and Penev, 1998; Cagli, 1984). Therefore, these indicators cannot be used to determine whether the improvement in the metric is due to model complexity or data fitting the model for nested models (Marsh et al., 2004). Nonetheless, following the methodology of the original version of the SCQ, we employed these indicators to determine the validity of the models in this study. This limitation is addressed in Study 2.

2 SUPPLEMENTARY RESULTS

2.1 Factor Structure

It was assumed that an improper solution was caused by a localized solution. Thus, we conducted further confirmatory analysis by excluding Item 47, which had a low factor loading in the model. However, improper solutions remained unchanged (Supplementary Table 5). Next, we performed the analysis after adding the constraint that the error variances were > 0, because the direct cause of improper solutions was that the error variance of the latent variable was less than 0. Improper solutions did not appear in this model (Supplementary Tables 6). However, strong correlations between factors were present, suggesting that the factor structure may not be appropriate (Supplementary Tables 7). Based on these results, we decided that the original model was not suitable for the present data.

2.2 Re-examination of the Factor Structure

As the confirmatory factor analysis produced improper solutions, we reanalyzed the acquired data using exploratory factor analysis, assuming a one-factor structure. In this case, the factor loadings of Items 46 and 47 fell below 0.200 (0.127 and 0.107, respectively; Supplementary Table 8). Therefore, we excluded these items from the Japanese version of the SCQ and alternatively added Item 49, which was included in the same factor in the SCQ-L. As a result, no items had factor loadings below 0.200 (Supplementary Table 9); we decided to use these items as the scale items for the Japanese version of the SCQ.

In addition to the two-factor structure, we examined the following two types of structural models: three-factor and nine-factor models. As a result, knowingness, attitude, and behavior factors were barely retained, but the original nine-factor structure was not markedly preserved (Supplementary table 10-11).

Note that the results of the exploratory factor analysis showed that the knowingness, attitude, and behavior factor items were regarded as relatively the same, but some items were included in different factors (Table 1, Supplementary Tables 10). However, this slight difference from the original version can be considered a result of the indeterminacy of the factor rotation and the different rotation methods chosen (See the discussion, Elffers et al., 1978).

3 REFERENCE

Baer, R. A., Smith, G. T., Lykins, E., Button, D., Krietemeyer, J., Sauer, S., Walsh, E., Duggan, D., and Williams, J. M. G. (2008). Construct validity of the five facet mindfulness questionnaire in meditating and nonmeditating samples. *Assessment* 15, 329–342. doi:10.1177/1073191107313003.

Berglund, T., Gericke, N., Boeve-de Pauw, J., Olsson, D., and Chang, T.-C. (2020). A cross-cultural comparative study of sustainability consciousness between students in Taiwan and Sweden. *Environment, Development and Sustainability* 22, 6287–6313. doi:10.1007/s10668-019-00478-2.

Brereton, R. G. (2015). The chi squared and multinormal distributions. *Journal of Chemometrics* 29, 9–12. doi:10.1002/cem.2680.

Buuren, S. van (2018). *Flexible imputation of missing data*. CRC Press, Taylor & Francis Group.

Cagli, U. (1984). Nested model comparison with structural equation approaches. *Journal of Business Research* 12, 309–318. doi:10.1016/0148-2963(84)90014-6.

Elffers, H., Bethlehem, J., and Gill, R. (1978). Indeterminacy problems and the interpretation of factor analysis results. *Statistica Neerlandica* 32, 181–199. doi:10.1111/j.1467-9574.1978.tb01398.x.

Gerbing, D. W., and Anderson, J. C. (1984). On the meaning of within-factor correlated measurement errors. *Journal of Consumer Research* 11, 572–580.

Little, R. (2019). *Statistical analysis with missing data., 3rd edition*. Wiley.

Little, T. D., Cunningham, W. A., Shahar, G., and Widaman, K. F. (2002). To parcel or not to parcel: Exploring the question, weighing the merits. *Structural equation modeling* 9, 151–173.

Marsh, H., Hau, K.-T., and Wen, Z. (2004). In search of golden rules: Comment on hypothesis-testing approaches to setting cutoff values for fit indexes and dangers in overgeneralizing Hu and Bentler's (1999) findings. *Structural Equation Modeling* 11, 320–341. doi:10.1207/s15328007sem1103_2.

Raykov, T., and Penev, S. (1998). Nested structural equation models: Noncentrality and power of restriction test. *Structural Equation Modeling: A Multidisciplinary Journal* 5, 229–246. doi:10.1080/10705519809540103.

Rocha, C., and Chelladurai, P. (2012). Item parcels in structural equation modeling: An applied study in sport management. *International Journal of Psychology and Behavioral Sciences* 2, 46–53. doi:10.5923/j.ijpbs.20120201.07.

Savalei, V., Brace, J., and Fouladi, R. T. (2021). We need to change how we compute RMSEA for nested model comparisons in structural equation modeling. doi:10.31234/osf.io/wprg8.

Tabachnick, B. G., and Fidell, L. S. (2013). *Using multivariate statistics*. Pearson.

Takahashi, T., Saito, J., Fujino, M., Sato, M., and Kumano, H. (2022). The validity and reliability of the short form of the five facet mindfulness questionnaire in Japan. *Frontiers in Psychology* 13.

Yuan, K.-H., and Bentler, P. M. (2000). 5. Three likelihood-based methods for mean and covariance structure analysis with nonnormal missing data. *Sociological Methodology* 30, 165–200. doi:10.1111/0081-1750.00078.

Supplementary Table 1 Item descriptions of the sustainability consciousness questionnaire.

Item Number	Item Code	Japanese	Back-translation from Japanese
SCQ_1*	K3 (ENV)+	水の消費量を減らすことは，持続可能な開発に必要だ。	Limiting our water consumption levels is necessary for sustainable development.
SCQ_2	K4i (ENV)	自然を守ることは，持続可能な開発に必要ない	Protecting nature is not necessary for sustainable development.
SCQ_3	K7 (ENV)	持続可能な開発には，私たち人間があらゆる種類の廃棄物を減らすことが必要だ。	Sustainable development requires that we humans reduce the generation of all kinds of waste.
SCQ_4*	K14 (ENV)+	生き物の多様性を守る（生物学的多様性を守る）ことは，持続可能な開発に必要である。	The protection of biological diversity (biodiversity) is necessary for sustainable development.
SCQ_5	K18 (ENV)	持続可能な開発は，再生可能な天然資源への転換を必要とする。	Sustainable development would result from a switch to renewable natural resources.
SCQ_6*	K21 (ENV)+	持続可能な発展のためには，人々は自然災害から身を守る方法について教育を受ける必要がある。	Sustainable development requires people to be educated about how to protect themselves from natural disasters.
SCQ_7	K2 (SOC)	人々が健康で長生きする機会を増やすことは，持続可能な開発に寄与する。	Increasing opportunities for people to lead long and healthy lives contribute to sustainable development.
SCQ_8*	K5 (SOC)+	話し合いを通して紛争を平和的に解決する文化が，持続可能な開発に必要である。	A culture of conflict resolution through peaceful discussion is necessary for sustainable development.
SCQ_9	K8 (SOC)	民主的な権利を行使する人々が，持続可能な開発に必要である（例えば，彼らが選挙に投票したり，社会問題に関与したり，彼らの意見を表明したりなど）。	People who exercise democratic rights are necessary for sustainable development (for example, they vote in elections, engage in social issues, express their opinions, etc.).

Continues

Continued

Item Number	Item Code	Japanese	Back-translation from Japanese
SCQ_10	K9 (SOC)	少女や女性の権利を強化し，世界中で平等性を高めることは，持続可能な開発に必要である。	Further assertion of women and girls rights and an increase in equality worldwide is necessary for sustainable development.
SCQ_11*	K10 (SOC)+	人権を尊重することは，持続可能な開発に必要である。	Respecting human rights is necessary for sustainable development.
SCQ_12*	K11 (SOC)+	持続可能な開発を達成するためには，世界中のすべての人々が良い教育を受けられるようにしなければならない。	To achieve sustainable development, everyone in the world must have access to quality education.
SCQ_13	K15 (SOC)	他の文化へ敬意を持つことは，持続可能な開発に必要である。	Respecting other cultures is necessary for sustainable development.
SCQ_14	K20 (SOC)	持続可能な開発のためには，HIV/AIDSやマラリアなどの深刻な感染症を阻止しなければならない。	The prevention of serious infectious diseases such as HIV/AIDS and malaria is essential for sustainable development.
SCQ_15*	K12 (ECO)+	持続可能な開発には，企業が従業員や顧客，仕入れ先に対して責任を持って行動することが必要だ。	Sustainable development requires corporations to act responsibly toward their employees, customers, and suppliers.
SCQ_16*	K16 (ECO)+	持続可能な開発には，世界の人々への財とサービスのフェアな分配が必要だ。	Sustainable development requires fair distribution of goods and services to people around the world.
SCQ_17*	K17 (ECO)+	世界の貧困をなくすことは持続可能な開発に必要である。	The eradication of poverty at an international level is necessary for sustainable development.
SCQ_18	K19 (ECO)	持続可能な開発には，どのように経済が動くか人々が理解する必要がある。	Sustainable development requires people to understand the functioning of the economy.

Continues

Continued

Item Number	Item Code	Japanese	Back-translation from Japanese
SCQ_19*	A5i (ENV)+	私たちが必要以上に天然資源を使っても，将来の人々の健康や幸福を脅かすことはないと思う。	I think using more natural resources than are necessary for sustenance will not threaten the health and happiness of future generations.
SCQ_20*	A6 (ENV)+	環境を守るには，より厳しい法律や規制が必要だと思う。	I think more stringent laws and regulations are necessary to protect the environment.
SCQ_21*	A10 (ENV)+	気候変動に関連した問題へ対策をとることは重要だと思う。	I believe that it is essential to address issues pertaining to climate change.
SCQ_22	A19i (ENV)	私たち一人一人が好きなだけ水を使ってもいいと思う。	I believe that each of us is free to use as much water as desired.
SCQ_23*	A1 (SOC)+	持続可能な生活をするために必要な知識や価値観，スキルを身につける機会を誰もが与えられるべきだと思う。	I think each individual should be provided with the opportunity to acquire the knowledge, values, and skills necessary to lead a sustainable life.
SCQ_24*	A2 (SOC)+	今生きている私たちは，未来の人々が今と同じクオリティの生活を享受できるようにすべきだと思う。	I believe that individuals of the present generation should ensure that the future generations can reap the benefits of the same quality of life that we enjoy today.
SCQ_25	A11 (SOC)	環境に良い車へより多くの人々が乗り換えるのを促進するために，政府は財政援助をすべきだと思う。	I think the government should provide financial aid to encourage more people to switch to eco-friendly cars.
SCQ_26	A13 (SOC)	政府は持続可能な開発を基準としてすべての意思決定をすべきだと思う。	I think the government should base all of its decisions on sustainable development.
SCQ_27	A14 (SOC)	社会の人々が民主的な権利を行使し，重大な問題に関与することが重要だと思う。	I think it is imperative for citizens to exercise their democratic rights and participate in the resolution of pressing social issues.

Continues

Continued

Item Number	Item Code	Japanese	Back-translation from Japanese
SCQ_28*	A18 (SOC)+	世界中の女性と男性が，教育や雇用に対する同等な機会が与えられるべきだと思う。	I believe that equal opportunities should be provided to women and men across the world in terms of education, employment, etc.
SCQ_29*	A3 (ECO)+	企業は梱包材の使用や使い捨て用品を減らす責任があると思う。	I think corporations shoulder the responsibility to regulate the use of packaging materials and disposable items.
SCQ_30*	A7 (ECO)+	貧困を減らすことは重要だと思う。	I believe that reducing the poverty levels is crucial.
SCQ_31*	A8 (ECO)+	豊かな国の企業は，貧しい国の従業員に自国と同じ条件を与えるべきだと思う。	I think corporations in rich countries should provide their employees based in economically underdeveloped countries with the same working conditions as those enjoyed by employees in the companies home country.
SCQ_32	A16 (ECO)	土地や空気，水を汚染した人々は，環境に与えた損害の対価を払うべきだと思う。	I think penalties on account of damaging the environment should be levied on individuals polluting soil, air, water, etc.
SCQ_33	B1(ENV)	どこかへ行くとき，可能であれば，車で移動する代わりに自転車や徒歩で移動することにしている。	I prefer to commute on foot or by bicycle rather than by car, if possible.
SCQ_34	B2 (ENV)	私は決して水を無駄にしない。	I never waste water.
SCQ_35*	B3 (ENV)+	私はできる限りリサイクルする。	I recycle as much as I can.
SCQ_36	B7 (ENV)	私は田舎や公共の場でゴミを見かけたら拾う。	When I come across garbage in public or rural areas, I pick it up.
SCQ_37	B8i (ENV)	私は，自分の行動がどのように自然環境に損害を与えうるかについて考えない。	I do not think about the ways in which my actions may damage the natural environment.

Continues

Continued

Item Number	Item Code	Japanese	Back-translation from Japanese
SCQ_38*	B10 (ENV)+	私は機会があると，ゴミを出す前に必ず分別をする。	Where possible, I always separate my garbage before putting it out.
SCQ_39*	B12 (ENV)+	廃棄物を減らすために，生活スタイルを変えた（例：食べ物をあまり捨てないようにする，ものを無駄にしない）。	I have made lifestyle changes to ensure waste reduction (i.e., by trying to avoid wastage of any kind, including that of food).
SCQ_40*	B4 (SOC)+	コンピュータや携帯電話を使ってチャットやメール，ゲームなどをするときは，いつも実生活と同じように相手を尊重している。	When I chat, email, play games, etc., on my computer or smartphone, I always treat others with the same respect as I would in the concrete world.
SCQ_41	B5i (SOC)	私は健康に良くないライフスタイルを選択することが多い。	I often make lifestyle choices that are harmful to my health.
SCQ_42	B13 (SOC)	私は学校で委員会（例えば，生徒会，クラス委員会）に所属している。	I belong to a committee at school (for example, the student committee, the class committee).
SCQ_43	B14 (SOC)	私は，たとえ自分とは異なる文化的背景を持っていても，すべての人に同じように敬意を払って接する。	I treat everyone with the same respect, including people whose cultural backgrounds are different from mine.
SCQ_44*	B15 (SOC)+	私は，援助団体もしくは環境保護団体を支援している。	I support aid organizations and/or environmental groups.
SCQ_45*	B17 (SOC)+	私は，男性にも女性にも，男子にも女子にも同じように敬意を払う。	I treat men and women as well as boys and girls with the same respect.
SCQ_46	B6 (ECO)+	私は，貧しい人々を支援する活動をしている。	I engage in activities to support individuals living in poverty.
SCQ_47	B9 (ECO)+	私はインターネットやお店で中古品を購入することが多い。	I often purchase second-hand goods online or in stores.

Continues

Continued

Item Number	Item Code	Japanese	Back-translation from Japanese
SCQ_48*	B11 (ECO)+	私は，従業員や環境への配慮で評判の悪い会社の商品は買わないようにしている。	I try not to purchase goods from companies that have bad reputations in terms of their cognizance toward the welfare of their workers and the environment.
SCQ_49*	B16 (ECO)	私は，経済に関するニュース番組を見るもしくは新聞記事を読む。	I watch news programs on the economy and/or read newspaper articles.

Note. All items in the long version of the SCQ (SCQ-L) were translated. The items selected for the Japanese version are marked with * after each item number. The Item Code indicates the item code of the original version. + indicates the item of the SCQ-S, and i indicates that the item is reversed. The characters in parentheses, i.e., ENV, SOC, and ECO, indicate the factor to which the item belongs. K: knowingness; A: attitudes; B: behavior; ECO: economic; SOC: social; ENV: environmental.

Supplementary Table 2. Descriptive statistics for the sustainability consciousness questionnaire items in Study 1.

Item Number	Item Code	*Mean*	*SD*	*Skewness*	*Kurtosis*
SCQ_1*	K3 (ENV)*	3.806	0.760	0.591	−0.491
SCQ_2i	K4i (ENV)	1.821	0.921	2.682	−1.521
SCQ_3	K7 (ENV)	4.186	0.630	−0.259	−0.245
SCQ_4*	K14 (ENV)*	4.189	0.643	−0.046	−0.324
SCQ_5	K18 (ENV)	4.041	0.634	0.014	−0.192
SCQ_6*	K21 (ENV)*	4.199	0.605	−0.480	−0.119
SCQ_7	K2 (SOC)	3.712	0.803	0.083	−0.321
SCQ_8*	K5 (SOC)*	4.169	0.676	−0.323	−0.327
SCQ_9	K8 (SOC)	3.900	0.645	−0.075	−0.137
SCQ_10	K9 (SOC)	4.020	0.715	0.676	−0.518
SCQ_11*	K10 (SOC)*	4.178	0.646	−0.065	−0.313
SCQ_12*	K11 (SOC)*	4.162	0.650	0.484	−0.460
SCQ_13	K15 (SOC)	4.149	0.632	−0.582	−0.128
SCQ_14	K20 (SOC)	4.105	0.687	1.181	−0.630
SCQ_15*	K12 (ECO)*	4.182	0.562	0.365	−0.088
SCQ_16*	K16 (ECO)*	3.742	0.715	−0.323	−0.069
SCQ_17*	K17 (ECO)*	4.050	0.711	0.989	−0.623
SCQ_18	K19 (ECO)	4.115	0.583	0.666	−0.224
SCQ_19i*	A5i (ENV)*	1.954	0.838	0.685	−0.830
SCQ_20*	A6 (ENV)*	3.669	0.837	−0.229	−0.370
SCQ_21*	A10 (ENV)*	4.332	0.646	1.770	−0.707
SCQ_22i	A19i (ENV)	2.086	0.843	1.009	−0.833
SCQ_23*	A1 (SOC)*	4.275	0.600	0.838	−0.468
SCQ_24*	A2 (SOC)*	3.871	0.807	0.146	−0.520
SCQ_25	A11 (SOC)	3.950	0.791	0.448	−0.635
SCQ_26	A13 (SOC)	3.285	0.842	−0.146	−0.008
SCQ_27	A14 (SOC)	3.927	0.636	−0.139	−0.090
SCQ_28*	A18 (SOC)*	4.355	0.609	0.305	−0.528
SCQ_29*	A3 (ECO)*	4.093	0.676	0.530	−0.499
SCQ_30*	A7 (ECO)*	4.342	0.589	−0.135	−0.347
SCQ_31*	A8 (ECO)*	3.493	0.818	−0.529	−0.035
SCQ_32	A16 (ECO)	3.736	0.794	0.065	−0.271
SCQ_33	B1 (ENV)	3.599	1.048	−0.285	−0.582
SCQ_34	B2 (ENV)	3.437	0.890	−0.640	−0.008
SCQ_35*	B3 (ENV)*	3.854	0.733	0.147	−0.425
SCQ_36	B7 (ENV)	2.983	0.887	−0.185	0.146
SCQ_37i	B8i (ENV)	2.709	0.941	−0.574	−0.367
SCQ_38*	B10 (ENV)*	4.286	0.656	0.254	−0.587
SCQ_39*	B12 (ENV)*	3.536	0.983	−0.358	−0.519
SCQ_40*	B4 (SOC)*	3.886	0.763	0.345	−0.492
SCQ_41i	B5i (SOC)	2.768	0.907	−0.637	−0.231
SCQ_42	B13 (SOC)	1.820	0.976	0.091	0.985
SCQ_43	B14 (SOC)	3.907	0.719	−0.094	−0.290
SCQ_44*	B15 (SOC)*	2.539	0.999	−0.698	0.138
SCQ_45*	B17 (SOC)*	4.182	0.613	−0.499	−0.123
SCQ_46	B6 (ECO)*	2.275	0.958	−0.452	0.426
SCQ_47	B9 (ECO)*	2.788	1.159	−0.927	0.225
SCQ_48*	B11 (ECO)*	3.377	0.884	−0.490	−0.174
SCQ_49*	B16 (ECO)	3.517	1.052	−0.127	−0.734

Note. The survey in Study 1 required responses to all the items of the long version of the SCQ. The items selected for the Japanese version are marked with * after each item number. The Item Code indicates the item number of the original version. the items of the short version of the SCQ are marked with * after each item code. ”i” indicates that the item is reversed. The characters in parentheses, i.e., ENV, SOC, and ECO, indicate the factor to which the item belongs. K: knowingness; A: attitudes; B: behavior; ECO: economic; SOC: social; ENV: environmental.

Supplementary Table 3. Factor loading of each item for nine subfactors.

Factor/Item	Factor Loading	*SE*
K_ENV		
SCQ_1	0.465	0.060
SCQ_4	0.543	0.058
SCQ_6	0.491	0.073
K_SOC		
SCQ_8	0.647	0.045
SCQ_11	0.725	0.036
SCQ_12	0.667	0.054
K_ECO		
SCQ_15	0.619	0.044
SCQ_16	0.514	0.048
SCQ_17	0.636	0.045
A_ENV		
SCQ_19i	0.519	0.055
SCQ_20	0.484	0.054
SCQ_21	0.713	0.042
A_SOC		
SCQ_23	0.523	0.063
SCQ_24	0.477	0.047
SCQ_28	0.564	0.054
A_ECO		
SCQ_29	0.625	0.041
SCQ_30	0.619	0.049
SCQ_31	0.474	0.056
B_ENV		
SCQ_35	0.629	0.064
SCQ_38	0.523	0.101
SCQ_39	0.585	0.065
B_SOC		
SCQ_40	0.297	0.083
SCQ_44	0.280	0.102
SCQ_45	0.370	0.099
B_ECO		
SCQ_46	0.443	0.111
SCQ_47	0.206	0.075
SCQ_48	0.491	0.097

Note. SE: Standard Error; K: knowingness; A: attitudes; B: behaviour; ECO: economic; SOC: social; ENV: environmental.

Supplementary Table 4. Factor correlation matrix of the original model.

	SC	K	A	B	K_ENV	K_SOC	K_ECO	A_ENV	A_SOC	A_ECO	B_ENV	B_SOC	B_ECO
SC													
K	0.938												
A	1.002	0.940											
B	0.754	0.708	0.756										
K_ENV	0.916	0.977	0.918	0.691									
K_SOC	0.877	0.935	0.879	0.662	0.914								
K_ECO	0.985	1.050	0.987	0.743	1.026	0.982							
A_ENV	0.866	0.813	0.864	0.653	0.794	0.760	0.853						
A_SOC	1.086	1.019	1.083	0.819	0.995	0.953	1.070	0.936					
A_ECO	1.018	0.955	1.015	0.768	0.933	0.893	1.003	0.878	1.100				
B_ENV	0.681	0.639	0.682	0.903	0.624	0.598	0.671	0.590	0.739	0.693			
B_SOC	1.214	1.139	1.217	1.610	1.113	1.066	1.197	1.052	1.318	1.236	1.453		
B_ECO	0.569	0.533	0.570	0.754	0.521	0.499	0.560	0.492	0.617	0.579	0.681	1.213	

Note. SC: sustainability consciousness, K: knowingness, A: attitudes, B: behavior, ECO: economic, SOC: social, ENV: environmental.

Supplementary Table 5. Factor loadings of the original model (item 47 was deleted).

Factor/Item	Factor Loading	*SE*
SC		
K	0.937	0.037
A	1.004	0.033
B	0.759	0.092
K		
K_ENV	0.977	0.068
K_SOC	0.935	0.034
K_ECO	1.050	0.037
A		
A_ENV	0.865	0.049
A_SOC	1.083	0.062
A_ECO	1.015	0.046
B		
B_ENV	0.909	0.072
B_SOC	1.587	0.361
B_ECO	0.751	0.192
K_ENV		
SCQ_1	0.465	0.060
SCQ_4	0.543	0.058
SCQ_6	0.491	0.073
K_SOC		
SCQ_8	0.647	0.045
SCQ_11	0.725	0.036
SCQ_12	0.667	0.055
K_ECO		
SCQ_15	0.620	0.044
SCQ_16	0.514	0.048
SCQ_17	0.636	0.045
A_ENV		
SCQ_19i	0.520	0.055
SCQ_20	0.484	0.054
SCQ_21	0.713	0.042
A_SOC		
SCQ_23	0.523	0.063
SCQ_24	0.477	0.047
SCQ_28	0.564	0.054
A_ECO		
SCQ_29	0.626	0.041
SCQ_30	0.618	0.049
SCQ_31	0.474	0.056
B_ENV		
SCQ_35	0.630	0.064
SCQ_38	0.529	0.100
SCQ_39	0.582	0.065
B_SOC		
SCQ_40	0.299	0.083
SCQ_44	0.278	0.102
SCQ_45	0.376	0.099
B_ECO		
SCQ_46	0.438	0.114
SCQ_48	0.490	0.100

Note. *SE*: Standard Error; SC: sustainability consciousness; K: knowingness; A: attitudes; B: behaviour; ECO: economic; SOC: social; ENV: environmental.

Supplementary Table 6. Factor loading of the original model (constraints were added).

Factor/Item	Factor Loading	*SE*
SC		
K	0.947	0.018
A	0.965	0.010
B	0.945	0.019
K		
K_ENV	0.952	0.015
K_SOC	0.967	0.010
K_ECO	0.957	0.012
A		
A_ENV	0.967	0.009
A_SOC	0.955	0.013
A_ECO	0.970	0.008
B		
B_ENV	0.951	0.016
B_SOC	0.958	0.015
B_ECO	0.780	0.323
K_ENV		
SCQ_1	0.470	0.059
SCQ_4	0.561	0.052
SCQ_6	0.513	0.062
K_SOC		
SCQ_8	0.639	0.045
SCQ_11	0.715	0.035
SCQ_12	0.648	0.054
K_ECO		
SCQ_15	0.660	0.039
SCQ_16	0.537	0.047
SCQ_17	0.667	0.044
A_ENV		
SCQ_19i	0.509	0.050
SCQ_20	0.459	0.055
SCQ_21	0.669	0.037
A_SOC		
SCQ_23	0.606	0.044
SCQ_24	0.472	0.056
SCQ_28	0.642	0.039
A_ECO		
SCQ_29	0.658	0.040
SCQ_30	0.625	0.044
SCQ_31	0.476	0.055
B_ENV		
SCQ_35	0.493	0.054
SCQ_38	0.564	0.057
SCQ_39	0.493	0.065
B_SOC		
SCQ_40	0.496	0.052
SCQ_44	0.279	0.084
SCQ_45	0.575	0.049
B_ECO		
SCQ_46	0.184	0.118
SCQ_48	0.424	0.177

Note. *SE*: Standard Error; SC: sustainability consciousness; K: knowingness; A: attitudes; B: behaviour; ECO: economic; SOC: social; ENV: environmental.

Supplementary Table 7. Factor correlation matrix of the original model (constraints were added).

	SC	K	A	B	K_ENV	K_SOC	K_ECO	A_ENV	A_SOC	A_ECO	B_ENV	B_SOC	B_ECO
SC													
K	0.947												
A	0.965	0.914											
B	0.945	0.895	0.912										
K_ENV	0.902	0.952	0.870	0.853									
K_SOC	0.916	0.967	0.884	0.866	0.921								
K_ECO	0.906	0.957	0.874	0.857	0.911	0.925							
A_ENV	0.933	0.884	0.967	0.881	0.842	0.855	0.845						
A_SOC	0.921	0.873	0.955	0.870	0.831	0.844	0.835	0.923					
A_ECO	0.935	0.886	0.970	0.884	0.844	0.857	0.848	0.937	0.926				
B_ENV	0.898	0.851	0.866	0.951	0.811	0.823	0.814	0.838	0.827	0.840			
B_SOC	0.905	0.858	0.873	0.958	0.817	0.830	0.820	0.844	0.834	0.847	0.910		
B_ECO	0.737	0.699	0.711	0.780	0.665	0.676	0.668	0.688	0.679	0.690	0.742	0.747	

Note. SC: sustainability consciousness, K: knowingness, A: attitudes, B: behavior, ECO: economic, SOC: social, ENV: environmental.

Supplementary Table 8. Component matrix for one-factor model

Item	Factor 1
SCQ_11	0.666
SCQ_17	0.633
SCQ_15	0.625
SCQ_21	0.620
SCQ_8	0.615
SCQ_30	0.611
SCQ_29	0.610
SCQ_28	0.604
SCQ_12	0.604
SCQ_23	0.572
SCQ_4	0.529
SCQ_38	0.524
SCQ_45	0.513
SCQ_16	0.511
SCQ_24	0.475
SCQ_6	0.474
SCQ_40	0.468
SCQ_31	0.465
SCQ_35	0.463
SCQ_19i	0.462
SCQ_1	0.459
SCQ_20	0.424
SCQ_39	0.414
SCQ_48	0.284
SCQ_44	0.238
SCQ_46	0.127
SCQ_47	0.107

Note. i indicates that the item is reversed.

Supplementary Table 9. Component matrix for one-factor model (the final Japanese version of the SCQ)

Item	Factor 1
SCQ_11	0.666
SCQ_17	0.632
SCQ_15	0.626
SCQ_21	0.622
SCQ_8	0.615
SCQ_29	0.612
SCQ_30	0.611
SCQ_28	0.605
SCQ_12	0.603
SCQ_23	0.572
SCQ_4	0.530
SCQ_38	0.525
SCQ_45	0.513
SCQ_16	0.508
SCQ_6	0.475
SCQ_24	0.474
SCQ_19i	0.466
SCQ_40	0.466
SCQ_35	0.464
SCQ_31	0.463
SCQ_1	0.457
SCQ_20	0.423
SCQ_39	0.414
SCQ_48	0.284
SCQ_44	0.233
SCQ_49	0.211

Note. i indicates that the item is reversed.

Supplementary Table 10. Rotated component matrix for three-factor model

Item	Factor 1	Factor 2	Factor 3
K10 (SOC)	0.740	0.035	-0.069
K17 (ECO)	0.705	-0.083	0.082
K11 (SOC)	0.635	0.032	-0.016
A7 (ECO)	0.622	0.018	0.038
K16 (ECO)	0.552	-0.072	0.085
A8 (ECO)	0.527	-0.172	0.182
K5 (SOC)	0.482	0.264	-0.056
K12 (ECO)	0.405	0.298	0.037
A18 (SOC)	0.404	0.337	-0.043
B4 (SOC)	0.368	0.239	-0.097
A2 (SOC)	0.318	0.077	0.206
K14 (ENV)	0.313	0.312	0.004
K21 (ENV)	0.290	0.184	0.111
K3 (ENV)	0.268	0.096	0.230
B10 (ENV)	-0.015	0.611	0.152
A5i (ENV)	0.034	0.594	-0.016
A10 (ENV)	0.220	0.422	0.172
A1 (SOC)	0.332	0.402	-0.067
A3 (ECO)	0.208	0.350	0.267
A6 (ENV)	0.094	0.262	0.236
B17 (SOC)	0.245	0.252	0.167
B12 (ENV)	0.030	0.097	0.563
B3 (ENV)	-0.060	0.297	0.535
B15 (SOC)	0.091	-0.196	0.527
B11 (ECO)	0.180	-0.134	0.381
B16 (ECO)	-0.020	0.020	0.378

Note. Item indicates the item code in the original version. See Table 1 for correspondence with items in the Japanese version. i indicates that the item is reversed. The characters in parentheses, i.e., ENV, SOC, and ECO, indicate the factor to which the item belongs. K: knowingness; A: attitudes; B: behavior; ECO: economic; SOC: social; ENV: environmental.

Supplementary Table 11. Rotated component matrix for nine-factor model

Item	Factor 1	Factor 2	Factor 3	Factor 4	Factor 5	Factor 6	Factor 7	Factor 8	Factor 9
A7 (ECO)	0.694	0.017	0.016	0.008	0.077	0.030	0.080	-0.009	-0.095
K17 (ECO)	0.572	0.091	-0.002	0.101	-0.032	0.031	0.054	0.049	0.129
A8 (ECO)	0.253	0.065	0.118	0.164	0.027	0.119	-0.016	-0.271	0.247
K11 (SOC)	0.000	0.859	0.001	-0.012	-0.007	0.000	0.009	0.005	-0.012
K10 (SOC)	0.253	0.258	0.174	-0.137	0.191	0.166	-0.089	0.022	0.191
A10 (ENV)	-0.017	-0.010	1.016	-0.002	-0.005	-0.010	0.004	-0.009	-0.006
A6 (ENV)	0.006	-0.050	0.071	0.611	-0.033	0.062	-0.026	-0.016	0.058
A3 (ECO)	0.138	0.114	0.086	0.484	0.055	-0.028	0.124	0.048	-0.137
K3 (ENV)	0.180	-0.082	0.165	0.327	-0.030	-0.014	0.025	0.046	0.200
A2 (SOC)	0.017	0.244	-0.030	0.317	0.132	0.143	0.044	-0.246	-0.006
B17 (SOC)	0.008	0.007	0.022	-0.016	0.763	0.001	0.038	-0.038	0.020
B4 (SOC)	0.217	-0.067	-0.063	-0.013	0.354	0.138	-0.090	0.238	0.072
K14 (ENV)	0.041	0.030	0.020	0.030	0.038	0.622	0.051	-0.011	-0.008
K21 (ENV)	0.037	0.132	0.043	-0.020	-0.083	0.308	0.189	0.176	0.123
K12 (ECO)	0.098	0.154	0.131	0.172	0.040	0.292	-0.012	0.024	0.015
A5i (ENV)	0.026	0.010	0.116	0.172	0.195	0.255	0.013	0.084	-0.328
B12 (ENV)	0.127	-0.018	0.048	-0.031	0.001	0.103	0.632	-0.016	0.002
B3 (ENV)	-0.090	0.027	0.050	0.181	0.156	-0.037	0.448	0.196	0.078
B15 (SOC)	0.076	0.141	0.007	0.045	0.036	-0.227	0.424	-0.099	0.112
B16 (ECO)	0.035	0.007	0.009	-0.049	0.002	0.097	0.407	-0.069	-0.003
A1 (SOC)	0.111	0.186	0.119	-0.026	-0.010	0.167	0.054	0.398	-0.026
K5 (SOC)	0.083	0.207	0.107	0.147	-0.026	0.171	-0.089	0.299	0.173
A18 (SOC)	0.242	0.145	0.134	0.102	0.256	-0.108	-0.073	0.266	-0.081
B10 (ENV)	-0.134	0.044	0.077	0.195	0.207	0.220	0.134	0.244	-0.098
B11 (ECO)	-0.094	-0.012	0.021	0.034	0.147	0.012	0.217	0.013	0.481
K16 (ECO)	0.156	0.186	-0.073	0.175	0.116	0.050	-0.044	0.054	0.274

Note. Item indicates the item code in the original version. See Table 1 for correspondence with items in the Japanese version. i indicates that the item is reversed. The characters in parentheses, i.e., ENV, SOC, and ECO, indicate the factor to which the item belongs. K: knowingness; A: attitudes; B: behavior; ECO: economic; SOC: social; ENV: environmental.

Supplementary Table 12. Factor loading of each item in the one-factor parceled model.

Factor/Item	Factor Loading	*SE*
SC		
K_ENV_P	0.679	0.041
K_SOC_P	0.771	0.034
K_ECO_P	0.793	0.030
A_ENV_P	0.617	0.042
A_SOC_P	0.767	0.034
A_ECO_P	0.753	0.032
B_ENV_P	0.574	0.048
B_SOC_P	0.582	0.047
B_ECO_P	0.328	0.058

Note. P for each factor item indicates that the corresponding data were parceled. *SE*: standard error; K: knowingness; A: attitudes; B: behavior; ECO: economic; SOC: social; ENV: environmental; Sus Cons: sustainability consciousness.

Supplementary Table 13. Factor loading of each item in the two-factor parceled model.

Factor/Item	Factor Loading	*SE*
KA		
K_ENV_P	0.680	0.041
K_SOC_P	0.775	0.034
K_ECO_P	0.803	0.029
A_ENV_P	0.619	0.042
A_SOC_P	0.768	0.035
A_ECO_P	0.759	0.032
B		
B_ENV_P	0.712	0.046
B_SOC_P	0.692	0.044
B_ECO_P	0.433	0.057

Note. P for each factor item indicates that the corresponding data were parceled. *SE*: standard error; K: knowingness; A: attitudes; KA: knowingness/attitudes; B: behavior; ECO: economic; SOC: social; ENV: environmental.

Supplementary Table 14. Factor loading of each item in the three-factor parceled model.

Factor/Item	Factor Loading	*SE*
K		
K_ENV_P	0.687	0.042
K_SOC_P	0.790	0.033
K_ECO_P	0.829	0.028
A		
A_ENV_P	0.642	0.042
A_SOC_P	0.790	0.034
A_ECO_P	0.779	0.031
B		
B_ENV_P	0.716	0.047
B_SOC_P	0.692	0.044
B_ECO_P	0.427	0.058

Note. P for each factor item indicates that the corresponding data were parceled. *SE*: standard error; K: knowingness; A: attitudes; B: behavior; ECO: economic; SOC: social; ENV: environmental.

Supplementary Table 15. Factor loading of each item in the two-factor hierarchical parceled model.

Factor/Item	Factor Loading	*SE*
SC		
KA	0.972	0.075
B	0.771	0.050
KA		
K_ENV_P	0.680	0.041
K_SOC_P	0.775	0.034
K_ECO_P	0.803	0.029
A_ENV_P	0.619	0.042
A_SOC_P	0.768	0.035
A_ECO_P	0.759	0.032
B		
B_ENV_P	0.712	0.046
B_SOC_P	0.692	0.044
B_ECO_P	0.433	0.057

Note. P for each factor item indicates that the corresponding data were parceled. *SE*: standard error; SC: sustainability consciousness; K: knowingness; A: attitudes; KA: knowingness/attitudes; B: behavior; ECO: economic; SOC: social; ENV: environmental.

Supplementary Table 16. Factor loading of each item in the three-factor hierarchical parceled model.

Factor/Item	Factor Loading	*SE*
SC		
K	0.925	0.037
A	0.973	0.033
B	0.771	0.044
K		
K_ENV_P	0.687	0.042
K_SOC_P	0.790	0.033
K_ECO_P	0.829	0.028
A		
A_ENV_P	0.642	0.042
A_SOC_P	0.790	0.034
A_ECO_P	0.779	0.031
B		
B_ENV_P	0.716	0.047
B_SOC_P	0.692	0.044
B_ECO_P	0.427	0.058

Note. P for each factor item indicates that the corresponding data were parceled. *SE*: standard error; SC: sustainability consciousness; K: knowingness; A: attitudes; B: behavior; ECO: economic; SOC: social; ENV: environmental.

Supplementary Table 17. Factor correlation matrix of the three-factor model.

	K	A	B
K	1	0.906	0.728
A	0.906	1	0.786
B	0.728	0.786	1

Note. K: knowingness; A: attitude; B: behavior.

Supplementary Table 18. Factor correlation matrix of the three-factor hierarchical model.

	SC	K	A	B
SC	1	0.916	0.989	0.794
K	0.916	1	0.906	0.728
A	0.989	0.906	1	0.786
B	0.794	0.728	0.786	1

Note. SC: sustainability consciousness; K: knowingness; A: attitude; B: behavior.

Supplementary Table 19. Factor loading of each item for the one-factor model.

Factor/Item	Factor Loading	*SE*
SC		
SCQ_1	0.465	0.057
SCQ_4	0.517	0.052
SCQ_6	0.465	0.063
SCQ_8	0.601	0.048
SCQ_11	0.642	0.045
SCQ_12	0.580	0.056
SCQ_15	0.640	0.046
SCQ_16	0.524	0.047
SCQ_17	0.617	0.049
SCQ_19i	0.436	0.057
SCQ_20	0.414	0.059
SCQ_21	0.604	0.039
SCQ_23	0.553	0.054
SCQ_24	0.516	0.050
SCQ_28	0.582	0.043
SCQ_29	0.633	0.046
SCQ_30	0.604	0.048
SCQ_31	0.464	0.056
SCQ_35	0.445	0.053
SCQ_38	0.491	0.054
SCQ_39	0.410	0.062
SCQ_40	0.430	0.059
SCQ_44	0.264	0.069
SCQ_45	0.502	0.053
SCQ_48	0.303	0.057
SCQ_49	0.214	0.067

Note. SE: Standard Error; SC: sustainability consciousness.

Supplementary Table 20. Factor loading of each item for the three-factor model.

Factor/Item	Factor Loading	*SE*
K		
SCQ_1	0.470	0.056
SCQ_4	0.521	0.053
SCQ_6	0.468	0.065
SCQ_8	0.603	0.050
SCQ_11	0.653	0.047
SCQ_12	0.598	0.056
SCQ_15	0.667	0.045
SCQ_16	0.549	0.047
SCQ_17	0.653	0.048
A		
SCQ_19i	0.434	0.061
SCQ_20	0.426	0.061
SCQ_21	0.614	0.040
SCQ_23	0.542	0.059
SCQ_24	0.530	0.050
SCQ_28	0.583	0.045
SCQ_29	0.669	0.046
SCQ_30	0.609	0.048
SCQ_31	0.483	0.058
B		
SCQ_35	0.569	0.063
SCQ_38	0.526	0.065
SCQ_39	0.537	0.066
SCQ_40	0.434	0.072
SCQ_44	0.396	0.080
SCQ_45	0.583	0.052
SCQ_48	0.377	0.060
SCQ_49	0.287	0.076

Note. *SE*: Standard Error; K: knowingness; A: attitudes; B: behavior.

Supplementary Table 21. Factor loading of each item for the two-factor hierarchical model.

Factor/Item	Factor Loading	*SE*
SC		
KA	1.057	0.090
B	0.734	0.077
KA		
SCQ_1	0.472	0.054
SCQ_4	0.510	0.053
SCQ_6	0.457	0.065
SCQ_8	0.590	0.049
SCQ_11	0.637	0.047
SCQ_12	0.585	0.056
SCQ_15	0.649	0.045
SCQ_16	0.527	0.047
SCQ_17	0.629	0.048
SCQ_19i	0.415	0.060
SCQ_20	0.419	0.059
SCQ_21	0.603	0.040
SCQ_23	0.535	0.058
SCQ_24	0.520	0.050
SCQ_28	0.570	0.045
SCQ_29	0.640	0.046
SCQ_30	0.605	0.049
SCQ_31	0.482	0.055
B		
SCQ_35	0.566	0.062
SCQ_38	0.520	0.067
SCQ_39	0.533	0.066
SCQ_40	0.439	0.071
SCQ_44	0.395	0.081
SCQ_45	0.579	0.051
SCQ_48	0.388	0.058
SCQ_49	0.288	0.076

Note. *SE*: Standard Error; SC: sustainability consciousness; KA: knowingness/attitudes; B: behaviour.

Supplementary Table 22. Factor loading of each item for the three-factor hierarchical model.

Factor/Item	Factor Loading	*SE*
SC		
K	0.916	0.036
A	0.989	0.030
B	0.794	0.053
K		
SCQ_1	0.470	0.056
SCQ_4	0.521	0.053
SCQ_6	0.468	0.065
SCQ_8	0.603	0.050
SCQ_11	0.653	0.047
SCQ_12	0.598	0.056
SCQ_15	0.667	0.045
SCQ_16	0.549	0.047
SCQ_17	0.653	0.048
A		
SCQ_19i	0.434	0.061
SCQ_20	0.426	0.061
SCQ_21	0.614	0.040
SCQ_23	0.542	0.059
SCQ_24	0.530	0.050
SCQ_28	0.583	0.045
SCQ_29	0.669	0.046
SCQ_30	0.609	0.048
SCQ_31	0.483	0.058
B		
SCQ_35	0.569	0.063
SCQ_38	0.526	0.065
SCQ_39	0.537	0.066
SCQ_40	0.434	0.072
SCQ_44	0.396	0.080
SCQ_45	0.583	0.052
SCQ_48	0.377	0.060
SCQ_49	0.287	0.076

Note. SE: Standard Error; SC: sustainability consciousness; K: knowingness; A: attitudes; B: behavior.
